# Targeting Multidrug-Resistant *Pseudomonas aeruginosa* from Companion Animals Using Bacteriophages

**DOI:** 10.3390/pathogens15101067

**Published:** 2026-10-08

**Authors:** Margarida Gomes, Patricia Boto, Manuela Oliveira, Luís Tavares, Eva Cunha

**Affiliations:** 1 CIISA—Centre for Interdisciplinary Research in Animal Health, Faculty of Veterinary Medicine, University of Lisbon, 1300-477 Lisbon, Portugal; margaridaggomess@gmail.com (M.G.); pa.boto@campus.fct.unl.pt (P.B.); ltavares@fmv.ulisboa.pt (L.T.); 2 Associate Laboratory for Animal and Veterinary Sciences (AL4AnimalS), 1300-477 Lisbon, Portugal; 3 cE3c—Centre for Ecology, Evolution and Environmental Changes & CHANGE—Global Change and Sustainability Institute, Faculdade de Ciências, Universidade de Lisboa, 1749-016 Lisboa, Portugal

**Keywords:** *Pseudomonas aeruginosa*, bacteriophage, antimicrobial resistance, planktonic cells, biofilm, dogs, cats

## Abstract

Antimicrobial resistance poses a major challenge to veterinary and human medicine, particularly in the context of difficult-to-treat infections caused by multidrug-resistant (MDR) bacteria. *Pseudomonas aeruginosa*, a WHO priority pathogen, is known for its resistance profile and ability to form biofilms. As an alternative to conventional antibiotics, bacteriophage therapy has gained attention due to its specificity for target bacteria, including those in biofilms, without disrupting the host microbiota. This study aimed to evaluate the antimicrobial potential of bacteriophages JG005 and JG024 against *P. aeruginosa* isolates obtained from companion animals, in their planktonic and biofilm forms. First, a total of 29 clinical isolates were characterized in terms of their antimicrobial resistance profile and biofilm-forming ability. Then, phage activity against planktonic cells was assessed using spot tests and time-kill assays, while antibiofilm activity was evaluated in a microplate assay. The results showed that 68.97% of the isolates were MDR and 41.38% were biofilm producers. JG005 and JG024 demonstrated activity against 58.62% and 65.52% of the isolates, respectively, with time-kill assays confirming strong growth inhibition. Antibiofilm activity was limited and observed after short phage exposure times. These findings support the potential of bacteriophages as a complementary strategy against resistant *P. aeruginosa* infections in animals.

## 1. Introduction

Antimicrobial resistance (AMR) represents a major global health threat, reducing the effectiveness of antibiotics and leading to difficult-to-treat infections. AMR extends beyond human health, also affecting the animal and environmental sectors, as bacteria recognize no boundaries. Specifically, the close contact between companion animals and humans creates opportunities for the transmission of antimicrobial-resistant bacteria, posing a significant risk to public health [1,2]. Considering the high relevance of AMR, the World Health Organization (WHO) has published the WHO Bacterial Priority Pathogens List, to guide R&D strategies focused on the prevention and control of resistant strains [3]. Among these pathogens, *Pseudomonas aeruginosa*, a Gram-negative bacterium classified as high priority pathogen, is of particular concern, as it is frequently associated with difficult to treat infections in both humans and animals [4,5]. *P. aeruginosa* is also a member of the ESKAPE group of pathogens, which also includes *Enterococcus faecium*, *Staphylococcus aureus*, *Klebsiella pneumoniae*, *Acinetobacter baumannii*, and *Enterobacter*. These microorganisms share key characteristics that contribute to the global burden of AMR, including the ability to adapt to and colonize healthcare environments and a broad repertoire of intrinsic and acquired resistance determinants, enabling them to persist as major causes of antimicrobial-resistant infections [6,7]. 

In veterinary settings, *P. aeruginosa* can cause a wide range of infections due to its opportunistic nature [5,8,9]. In companion animals, *P. aeruginosa* has been associated with otitis, ulcerative keratitis, pyoderma, urinary tract infections, skin and wound infections and respiratory tract infections [5,8]. Moreover, studies have shown that *P. aeruginosa* isolates from dogs and cats exhibit high levels of resistance, particularly to fluoroquinolones, β-lactam and aminoglycosides, which could have significant implications for infection treatment [5,8,9,10]. Moreover, the treatment of *P. aeruginosa* infections in animals can also be impaired by the expression of virulence factors, such as biofilm formation, which may contribute to increasing the severity of these infections [11,12].

Given the rise of AMR, the development of alternative antimicrobial strategies is of great importance, with bacteriophage therapy emerging as one of the most promising approaches [13,14]. Bacteriophages are viruses that infect bacteria with high specificity, without disrupting the host microbiota. Depending on their life cycles, they can be classified as lytic or lysogenic [13,14]. Lytic phages are the most promising as therapeutic agents, being able to replicate within the host cell, leading to bacterial cell lysis [14]. The antimicrobial activity of these viruses against several bacterial species, including multidrug-resistant (MDR) pathogens of animal origin, has already been reported [15,16]. They exhibit high specificity, can self-replicate in the presence of their bacterial host, and are non-toxic to animals and plants, which may explain the lack of adverse side effects [17]. To date, the use of phages as a therapeutic approach remains limited, partly due to regulatory challenges; however, several studies have reported successful phage treatment in both humans and animals [13,18,19,20]. Specifically, the use of phage therapy for the treatment of *P. aeruginosa* otitis in dogs has already been reported, with promising results [16,18,19].

Considering the high antimicrobial potential of phages, this study aimed to evaluate the activity of bacteriophages JG005 and JG024 against planktonic cells and biofilms of *P. aeruginosa* isolates associated with different infections in dogs and cats.

## 2. Materials and Methods

### 2.1. Bacterial Collection and Growth Conditions

From 2019 to 2023, a total of 29 *P. aeruginosa* isolates were collected at the Laboratory of Bacteriology of the Faculty of Veterinary Medicine, University of Lisbon. They were obtained from clinical samples collected during routine procedures by trained veterinarians, following standard routine procedures after the owners’ consent, and not requiring ethics approval. All animals were cared for according to the rules set out by the current EU (Directive 2010/63/EC) and national (DL 113/2013) legislation and by the competent authority (Direção Geral de Alimentação e Veterinária, DGAV, https://www.dgav.pt/animais/, accessed on 29 July 2026) in Portugal, and no animal experiments were performed.

The clinical samples were obtained from cats and dogs with several types of infection, including skin infections (dermatitis and suture-related infections), urinary tract infections, reproductive infections, and others (spleen abscess and infections associated with medical devices such as esophageal tubes and orthopedic screws).

For the study, the isolates were propagated in Luria Broth (LB) medium (VWR, Leuven, Belgium) at 37 °C for 24 h. Additionally, four reference strains were included as controls: *P. aeruginosa* DSM 19880, *P. aeruginosa* DSM 19882, *P. aeruginosa* ATCC^®^ 27853™ and *Escherichia coli* ATCC^®^ 25922™. 

### 2.2. Antimicrobial Susceptibility Testing (AST) and Resistance Profile Characterization 

The antimicrobial susceptibility profile of the isolates under study was evaluated using the disc diffusion technique, as described in the CLSI Guidelines, supplement VET01S and M100 [21,22].

The antibiotics tested (Oxoid, Hampshire, UK) were chosen based on their common use in veterinary medicine and their relevance for public health: amikacin (30 µg), carbenicillin (100 µg), cephalexin (30 µg), cefoperazone (30 µg), cefovecin (30 µg), ceftazidime (30 µg), ciprofloxacin (5 µg), doxycycline (30 µg), enrofloxacin (5 µg), gentamicin (10 µg), marbofloxacin (5 µg), minocycline (30 μg), nitrofurantoin (300 μg), ofloxacin (5 µg), piperacillin (100 µg), streptomycin (10 μg), and tobramycin (10 μg). The antimicrobial panel tested against each isolate was selected according to the animal species, isolate characteristics, and clinical relevance. According to Magiorakos et al. (2012), AST results were used to classify isolates as MDR when they were non-susceptible to at least one agent from at least three antimicrobial categories [23]. Only acquired resistance was considered for the MDR categorization of the isolates. 

The multiple antibiotic resistance (MAR) index was also determined for each isolate using the formula: MAR Index = Nº. antimicrobials to which isolates tested resistant / Nº. antimicrobials tested [24]. 

### 2.3. Biofilm Formation Characterization

The ability of *P. aeruginosa* isolates to form biofilms was evaluated using the Red Congo Agar Method [25]. This method involves the propagation of the isolates on Congo Red agar (CRA) plates, containing BHI agar medium (VWR, Leuven, Belgium) supplemented with 5% sucrose (Millipore^®^ Sigma-Aldrich, Ontario, ON, Canada) and 0.08% Red Congo dye (Sigma-Aldrich, St. Louis, MO, USA).

The isolates were inoculated on CRA plates and incubated aerobically for 72 h at 37 °C. Biofilm formation was assessed and registered after 24 h, 48 h, and 72 h of incubation. Isolates that produced biofilm developed black dry crystalline colonies, while colonies from non-producing isolates remained pink or red colored [25,26]. *P. aeruginosa* ATCC^®^ 27853™ and *E. coli* ATCC^®^ 25922™ were used as positive and negative controls, respectively. Experiments were conducted in triplicate on independent days.

### 2.4. Bacteriophage Propagation, Stock Preparation and Titration

The bacteriophages *Pseudomonas* Phage DSMZ 19872 (JG005) and *Pseudomonas* Phage DSMZ 22045 (JG024), available from the Leibniz Institute DSMZ-German Collection of Microorganisms and Cell Cultures (Braunschweig, Germany), were selected for this study. Phages were obtained as ready-to-use phage suspensions, with titers ranging from 1 × 10^8^ to 1 × 10^11^ plaque-forming units (PFUs)/mL, which were then propagated to be used during the experiments. The recommended bacterial host strains *P. aeruginosa* DSM-19880 (specific host for the JG005 phage) and *P. aeruginosa* DSM-19882 (specific host for the JG024 phage) were used for phage propagation [27]. To determine the concentration of bacteriophages after propagation, phage titration was performed using the double agar overlay plaque assay [28]. The titer of the phage suspension was determined using the following formula: number of plaques × 10 × reciprocal of counted dilution = PFU/mL. Only phage suspensions that presented a titration of 10^9^ PFU/mL or higher were used.

### 2.5. Bacteriophage Host Range

To test the antimicrobial activity of both JG005 and JG024 bacteriophages against the bacterial isolates, an adaptation of the bacteriophage spot-on-lawn procedure was used [29,30]. The procedure involved adding 200 µL of a suspension of each isolate at 0.5 McFarland to 3 mL of molten LB top agar (0.75% agar), which was placed over a LB agar plate. After solidification, 5 µL of each bacteriophage stock solution was spotted on the bacterial lawn of each isolate. The plates were then incubated overnight at 37 °C. After incubation, specific bacteriophage-susceptible isolates showed clear areas (plaques of lysis) where the bacteriophage suspensions were spotted.

*P. aeruginosa* DSM 19880 and *P. aeruginosa* DSM 19882 were used as positive controls for bacteriophage JG005 and JG024, respectively.

### 2.6. Bacteriophage Activity Against Planktonic Cells

To determine the in vitro activity of the bacteriophages against planktonic cells, a kinetic time-kill assay (TKA) was conducted using a modified protocol [30]. The reference strains *P. aeruginosa* DSM 19880 and *P. aeruginosa* DSM 19882 were tested as positive controls, along with the bacterial isolates that were susceptible to one or both phages in the spot-on-lawn tests.

The assays were performed in 96-well flat-bottomed polystyrene microtiter plates (VWR, Leuven, Belgium), using bacterial suspensions with 5 × 10^7^ CFU/mL in LB broth (VWR, Leuven, Belgium). The positive control wells contained 100 µL of the corresponding bacterial suspensions + 100 µL of LB broth. The test wells contained 100 µL of the bacterial suspension + 100 µL of the specific bacteriophage suspension (yielding a final concentration of 5 × 10^7^; 5 × 10^8^ and 5 × 10^9^ PFU/mL), corresponding to multiplicities of infection (MOI) of 1, 10 and 100 respectively. The MOI was defined as the input ratio of phage particles to bacterial cells at the beginning of each assay and was calculated based on the initial bacterial concentration and phage titer.

Two types of negative controls were included in the assays: the blank negative control, containing only 200 µL of LB broth, and the negative control with the phages, consisting of 100 µL of LB broth + 100 µL of the specific bacteriophage suspension at a MOI of 1, 10 and 100.

The microplate was incubated for 24 h at 37 °C, during which the optical density at 600 nm (OD600) of the suspensions in the wells was obtained automatically every hour using the FLUOstar OPTIMA microplate reader (BMG LABTECH, Ortenberg, Germany).

For each condition, three technical replicates were included in each of one the three independent experiments. For each independent experiment, the mean OD600 of the three technical replicates was corrected by subtracting the mean OD600 of the three corresponding blank wells. This resulted in three independent OD600 values (n = 3) per isolate and condition, which were used for the statistical analysis. Technical replicates were therefore not treated as independent observations. 

### 2.7. Bacteriophage Activity Against Established Biofilms

To examine the activity of the bacteriophages against established biofilms, a modified protocol based on Mendes et al. (2014) and Pettit et al. (2005) was used [30,31]. The reference strains *P. aeruginosa* DSM 19880 and *P. aeruginosa* DSM 19882 were tested, along with the biofilm-producing isolates that were inhibited by one or both phages in the spot-on-lawn tests.

A bacterial suspension of 5 × 10^6^ CFU/mL was prepared in LB broth supplemented with 0.25% glucose (Merck, Darmstadt, Germany). Then, 100 µL of this suspension was added to the wells of two 96-well flat-bottom polystyrene microtiter plates (VWR, Leuven, Belgium) and incubated aerobically at 37 °C for 24 h to allow for biofilm formation. After incubation, the planktonic bacteria were carefully removed using sterile pipettes and the wells were gently washed with sterile saline. Then, medium or bacteriophage were added to the wells as follows: the positive control wells contained the established biofilm and 150 µL of LB broth supplemented with 0.25% glucose; and the test wells contained the established biofilm and 150 µL of the specific bacteriophage suspension diluted in LB medium supplemented with 0.25% glucose with PFU corresponding to MOI of 1, 10 and 100.

Each assay also included two negative controls: the blank negative control, containing only 150 µL of LB broth supplemented with 0.25% glucose, and the negative control with the phages, consisting of 150 µL of the specific bacteriophage suspension diluted in LB supplemented medium at a MOI of 1, 10 and 100.

After preparation, the microplates were incubated at 37 °C for either 4 or 24 h. At each time point, the biofilms in the wells were evaluated using AlamarBlue (AB) (G-Biosciences, St. Louis, MO, USA) according to previously described protocols (Mendes et al., 2014; Pettit et al., 2005), by adding 5 μL of AB to the wells after incubation, followed by gentle agitation at 100 rpm and incubation for 1 h at 37°C. Then, the microplates were shaken and the absorbances at 570 nm (OD570) and 600 nm (OD600) were measured using a microplate reader. The experiments were performed in triplicate on three independent assays.

For each condition, the biofilm susceptibility results were measured as the percent mean biofilm inhibition ± standard deviation. This value was determined using the formula provided by the manufacturer (Equation (1)), with one exception: the medium-only negative control in the formula was replaced by a more robust negative control that consisted of medium plus bacteriophage at each MOI (1, 10 and 100).

Strong antimicrobial suppression was defined as a ≥ 50% biofilm reduction when compared with the positive control [30].

Equation (1) formula used for the determination of the percentage of phage inhibition for each isolate:

(1) $$100-\left(\frac{(117216)A\lambda_{1}-\left(80586\right)A\lambda_{2}}{\left(155677\right)A^{\prime }\lambda_{2}-\left(14652\right)A^{\prime }\lambda_{1}}\times 100\right)$$

where 117216 = the molar extinction coefficient of AB in the oxidized form (blue) at 600 nm, 80586 = the molar extinction coefficient of AB in oxidized form at 570 nm, 155677 = the molar extinction coefficient of AB in the reduced form (pink) at 570 nm, 14652 = the molar extinction coefficient of AB in reduced form at 600 nm, A = the absorbance of test wells, A′ = the absorbance of negative control wells, λ1 = 570 nm, and λ2 = 600 nm.

### 2.8. Statistical Analysis

Statistical analysis of data was carried out using Microsoft Excel 2016^®^ and RStudio^®^ with the lme4, lmerTest, and emmeans R packages (Boston, MA, USA) [32,33,34]. Quantitative variables are expressed as mean values ± standard deviation. 

To account for the repeated measurements obtained from the same isolate and to appropriately model the dependence among observations within isolates, bacterial growth (TKA) and biofilm reduction data were analyzed using linear mixed-effects models, with the isolate included as a random intercept [35]. For the TKA data, the treatment group (Control, MOI 1, MOI 10, and MOI 100) was included as a fixed effect. The overall effect of the treatment group was assessed using a Type III ANOVA with Satterthwaite’s approximation for the degrees of freedom [33]. Dunnett’s post hoc test was used to compare each MOI with the control [34].

For the biofilm reduction evaluation, MOI (1, 10, and 100) and exposure time (4 h and 24 h) were included as fixed effects, together with their interaction. Post hoc pairwise comparisons between MOIs at each time point were adjusted using Tukey’s method, while comparisons between timepoints at each MOI were adjusted using the Holm method [34]. Adjusted *p*-values ≤ 0.05 were considered statistically significant.

Moreover, to quantify the overall antibacterial effect of each bacteriophage treatment, the area under the curve (AUC) was calculated for each 24 h time-kill curve using Python. AUC values were determined for the positive control and for each phage treatment at MOI 1, MOI 10, and MOI 100, based on OD600 measurements collected hourly from 0 to 24 h. The results are expressed as the percentage of AUC inhibition, presented as a descriptive summary of the overall growth dynamics.

## 3. Results

### 3.1. Bacterial Collection, Antimicrobial Susceptibility Profile and Biofilm Formation Ability Characterization 

**Figure 1:**
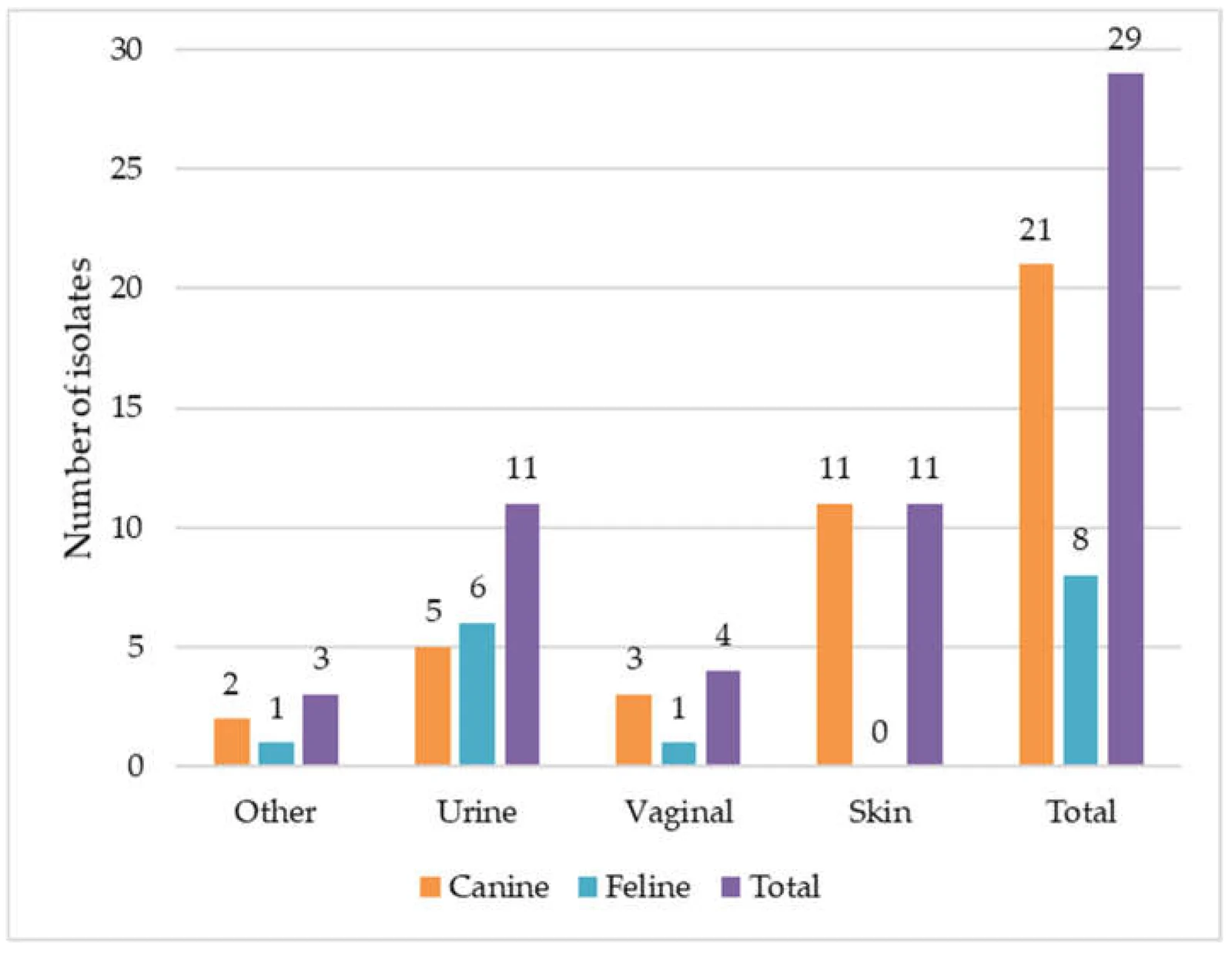
Distribution of the number of isolates by type of sample and animal species.

The bacterial collection under study included isolates from 21 dogs and 8 cats. The majority were obtained from samples of animals with skin (n = 11, 37.93%) and urinary tract (n = 11, 37.93%) infections, followed by reproductive tract infections (n = 4, 13.79%) and others (n = 3, 10.34%). The distribution of the number of isolates by type of sample and animal species is presented in Figure 1. 

The AST profile of the isolates is presented in Table 1. Considering the clinical presentation and animal species, the antimicrobials included in the AST for each isolate varied. Full data on the antimicrobials tested by each isolate can be observed in Supplementary File S1.

Isolates presented higher resistance regarding cefovecin, minocycline, doxycycline and nitrofurantoin, followed by cephalexin (88.46%) and streptomycin (85.71%), as shown in Table 1. Moreover, 68.97% (n = 20/29) of the *P. aeruginosa* isolates under study were classified as MDR.

The MAR index of the bacterial collection ranged from 0 to 0.67, with a mean value of 0.29 ± 0.18. Specifically, 41.38% (n = 12/29) of the isolates had a MAR index higher than 0.30.

A total of 12 isolates (12/29; 41.38%) were able to produce biofilm, 9 of which were originated from dogs and 3 from cats. Among these, 83.33% (n = 10/12) produced biofilm after 24 h of incubation, while 16.67% (n = 2/12) did so after 72 h of incubation.

### 3.2. Bacteriophage Host Range and Activity Against Planktonic Cells

**Figure 2:**
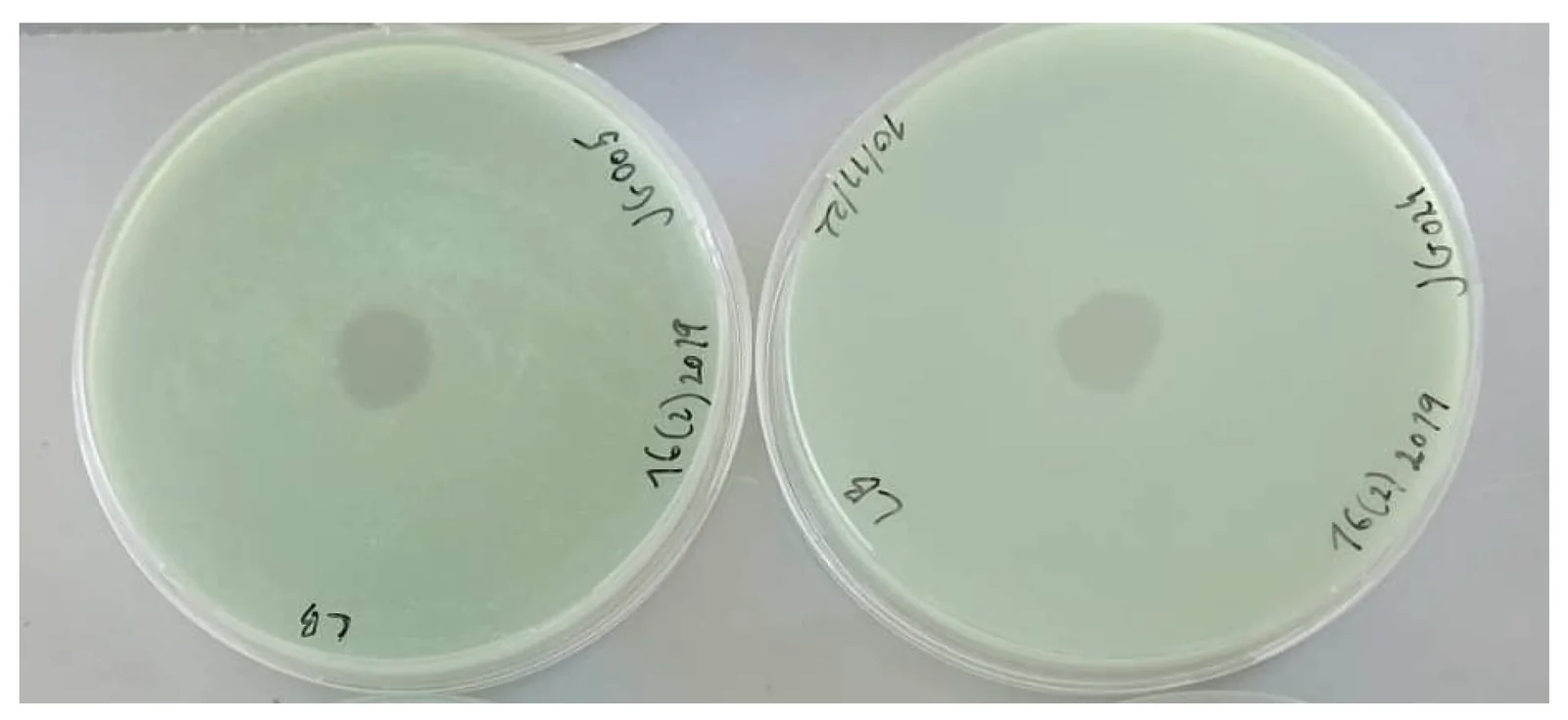
Spot-on-lawn tests for bacteriophages JG005 (**left**) and JG024 (**right**) against the *P. aeruginosa* isolate 16/19-2. The clear area on both plates demonstrates that the isolate is sensitive to both phages.

A total of 17 isolates (58.62%) were susceptible to phage JG005, 19 (65.52%) to phage JG024, and 15 (51.72%) to both bacteriophages (Figure 2, Table 2).

All susceptible isolates were further evaluated using a TKA, in which each isolate was exposed to the corresponding phage at MOIs of 1, 10, and 100. For each tested isolate, the OD600 values were measured and recorded after 24 h of exposure; Table 3 and Table 4 show the results regarding the phages JG005 and JG024, respectively. The corresponding time-kill curves for each isolate can be found in Supplementary File S2. 

The results of TKA revealed a reduction in bacterial growth in several isolates following phage exposure. JG005 inhibited the growth of all isolates except one (isolate 1086/20-1 1) at all the MOIs tested (1, 10 and 100), being able to inhibit 94.12% (n = 16/17) of the isolates. The growth of isolate 1086/20-1 1 was only inhibited at MOI 100 (Table 3). Overall, bacterial growth was significantly reduced at all three MOIs (*p* < 0.0001) when compared with the positive control, with the higher reduction observed at MOI 100 (Table 5). Regarding phage JG024, 94.74% (n = 18/19) of the isolates were inhibited after exposure at all MOIs. Only one isolate (626/19-1 3(1)) was not inhibited at MOI 100. The maximal growth suppression was observed when the isolates were exposed to MOI 1 (Table 4). Across the bacterial collection, the statistical analysis confirmed significantly (*p* < 0.0001) lower bacterial growth at all three MOIs relative to the positive control, with the higher estimated reduction being observed at MOI 1 (Table 6). Regarding the reference strains, the inhibition curves following exposure to the corresponding susceptible phages at MOIs of 1, 10, and 100 are presented in Figures S18 and S38 of Supplementary File S2, as well as the bacterial growth control curves. Both strains were inhibited by the specific phage at all MOIs, with higher inhibition observed when the phages were applied at MOI 1.

AUC analysis confirmed that both phages reduced bacterial growth across the susceptible isolates tested, while also revealing differences in the extent and persistence of inhibition among MOIs and between phages (Supplementary File S3). For JG005, the MOI 100 generally produced the strongest overall inhibition, being the best-performing condition towards most isolates. For JG024, the strongest inhibition was more variably distributed between MOI 1 and MOI 100.

### 3.3. Bacteriophage Activity Against Established Biofilms

The action of JG005 was tested using three biofilm-producing isolates and the phage reference strain. One isolate showed strong suppression at all MOIs after a four-hour incubation (MOI 1 = 73.14% ± 6.12; MOI 10 = 62.34% ± 11.25; MOI 100 = 69.32% ± 12.16).

**Figure 3:**
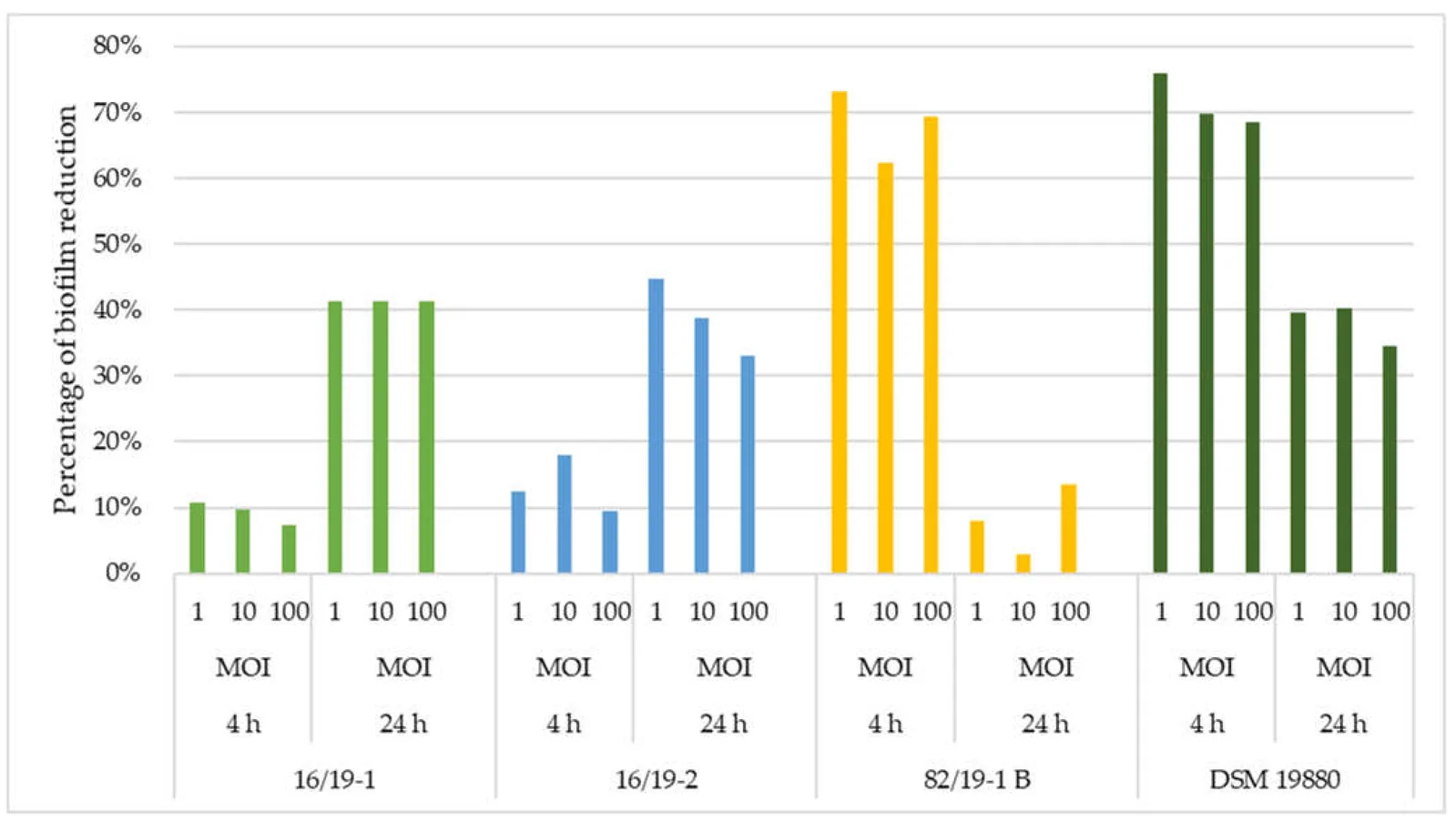
Percentage of biofilm reduction in four isolates (isolates 16/19-1, 16/19-2, 82/19-1 and the reference strain *P. aeruginosa* DSM 19880) after exposure to bacteriophage JG005 at different MOIs after 4 and 24 h.

The linear mixed-effects model and post hoc comparisons showed no statistically significant effects of MOI, exposure time, or their interaction on biofilm reduction (*p* > 0.05; Table 7 and Table 8), despite some variability among isolates. Individual isolate responses are shown in Figure 3.

Regarding the reference strain, strong suppression was found at all MOIs after four hours of incubation (Figure 3).

**Figure 4:**
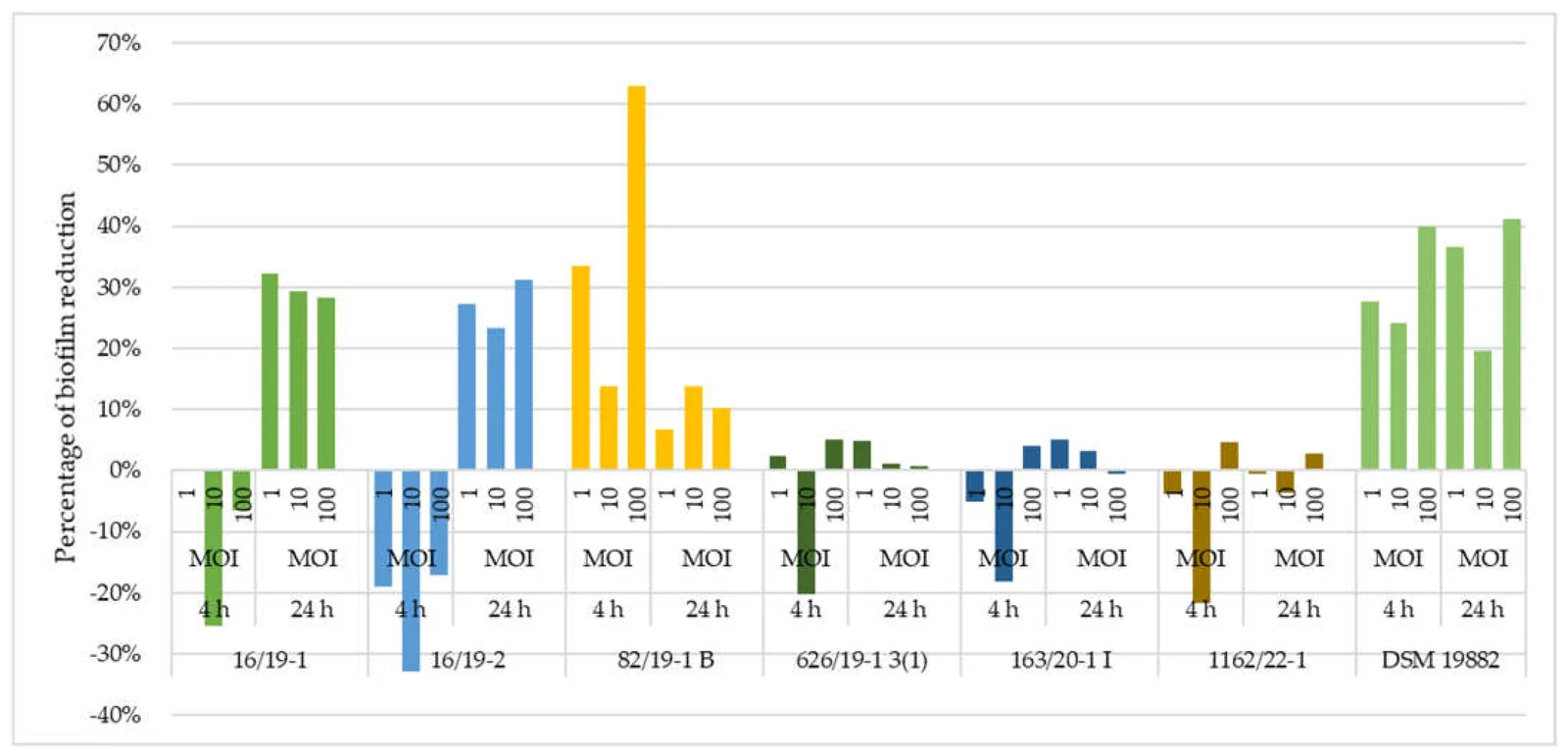
Percentage of biofilm reduction in seven isolates (isolates 16/19-1, 16/19-2, 82/19-1, 626/19-1 (3)1, 163/20-1, 1162/22-1 and the reference strain *P. aeruginosa* DSM 19882) after exposure to bacteriophage JG024 at different MOIs after 4 and 24 h. Negative percentage values represent increased biofilm formation.

The activity of JG024 was tested using 6 biofilm-producing isolates and the phage reference strain. As observed for bacteriophage JG005, one isolate showed strong suppression at MOI 100 after a four-hour incubation (63 ± 5.81%) (Figure 4). The linear mixed-effects model showed significant main effects of MOI (*p* = 0.031) and exposure time (*p* = 0.009), but no significant MOI × exposure time interaction (*p* = 0.211). Post hoc comparisons showed that biofilm reduction was significantly greater at MOI 100 than at MOI 10 after 4 h of exposure (*p* = 0.007), whereas this difference was no longer observed after 24 h (Table 9). In addition, biofilm reduction at MOI 10 increased significantly from 4 to 24 h (*p* = 0.005), whereas no significant changes over time were observed at MOI 1 or MOI 100 (Table 10). 

## 4. Discussion

*P. aeruginosa* is a bacterial pathogen that can cause infections in both humans and animals, being known for its ability to develop resistance to multiple antibiotics [4,5]. Bacteriophage therapy has been shown to have the potential to treat several bacterial infections in both human and veterinary medicine [36,37]. This study investigated the ability of two bacteriophages to inhibit 29 *P. aeruginosa* veterinary clinical isolates, in both planktonic and biofilm forms. 

As described for humans, *P. aeruginosa* acts as an opportunistic pathogen in various animal species, causing auricular, ocular, urogenital, wound, respiratory, and skin infections [5,8,9]. The bacterial collection used in this study mainly originated from skin and urinary tract infections in dogs, which is consistent with previous reports on *P. aeruginosa* infections in dogs and cats [38,39,40].

Regarding the antimicrobial profile of the isolates, high resistance rates to nitrofurantoin, cephalexin, carbenicillin, and enrofloxacin were observed. Li et al. (2021) reported resistance to cephalexin and nitrofurantoin in more than 20% of *Pseudomonas* spp. isolates in a study focusing on cats and dogs of the Iberian Peninsula [41], and Scott et al. (2019) reported a percentage of resistance to enrofloxacin of 33% in *P. aeruginosa* isolates from companion animals in the UK [42]. The high cephalexin, minocycline and doxycycline resistance observed is consistent with the intrinsic resistance of *P. aeruginosa* to this antimicrobial, being of limited clinical significance. Based on the definitions by Magiorakos et al. (2012) and Sweeney et al. (2018), 68.97% of the isolates under study were classified as MDR, which is in accordance with Nolff et al. (2016), who reported a percentage of MDR *P. aeruginosa* isolates from companion animals of 78% [23,43,44].

### 4.1. Phage Activity in Planktonic Cells

The two phages used in this study, JG005 and JG024, are Pbunaviruses belonging to the class Caudoviricetes [27,45,46], known for their broad antibacterial spectrum. Of the isolates tested, 58.62% were susceptible to phage JG005, 65.52% to phage JG024, and 51.72% to both phages in the spot test. These findings are consistent with those reported by Lourenço et al. (2025), who reported that 38.8% of canine otitis *P. aeruginosa* isolates were susceptible to JG005 and 61.2% to JG024 [16]. Similar results were also obtained by Furusawa et al. (2016) and De Melo et al. (2019), who tested other phages against *P. aeruginosa* isolates, observing antimicrobial activity against 46.15% and 51.4% of the tested strains, respectively [47,48]. Further characterization of the host range could be complemented by plaque assays and efficiency of plating, providing additional information on the productive infection of susceptible isolates. Following this initial screening, our study focused on a kinetic evaluation of phage activity using time-kill assays in microplates.

The TKA findings indicate a general trend toward bacterial reduction following phage exposure, although the response was heterogeneous and dependent on both the isolate and MOI. JG005 inhibited the growth of all isolates, at all MOIs, with the exception of one isolate (1086/20-1 1), which was only inhibited at MOI 100. Overall, maximal growth suppression was observed when bacteria were exposed to the highest phage concentration (MOI 100). Similar results were obtained by Sharma et al. (2021), using a *Pseudomonas* phage DRL-P1 belonging to the Myoviridae family. In that study, an eight-hour TKA using phage suspensions at MOIs of 100, 10, 1, 0.1, 0.01, and 0.001 showed that they inhibited bacterial growth, with higher MOIs being associated with lower bacterial growth [49]. In addition, the studies by Guo et al. (2019) and Abdelghafar et al. (2023), performed using other *P. aeruginosa* phages, reported that bacterial lysis was MOI dependent, with stronger inhibition at higher MOIs [50,51]. Our findings concerning JG005 are consistent with these studies, as they also show that, while bacterial growth was inhibited by different MOIs, MOI 100 was the most effective. In contrast, the results for JG024 differ from those reports, as the lowest MOI tested was the most effective. 

The differences observed across MOIs, both at the 24 h timepoint and in the overall percentage of growth inhibition based on AUC, support the idea that phage efficacy is not directly proportional to the initial phage dose, as infection dynamics, including adsorption efficiency and phage amplification following initial infection, may play a key role in bacterial reduction [52]. Although the linear mixed-effect models confirmed a significant overall reduction in bacterial growth across the bacterial collection for both phages, the global interpretation of efficacy should consider the variability in the magnitude of this response between isolates, as not all isolates responded equally to phage exposure. Some experimental variability was also observed between replicates, particularly for certain isolate–MOI combinations, which may reflect differences in phage–bacterium interactions and lysis kinetics, as well as the possible emergence of phage-insensitive or phage-resistant bacterial subpopulations. Overall, both JG005 and JG024 showed relevant antimicrobial activity; however, reduced efficacy was observed towards some isolates after approximately 12 h incubation, as indicated by an increase in OD600, with some values approaching those of the control group after 21 h. Unlike the findings published by Guo et al. (2019), this late increase was not exclusively associated with the highest MOI. The emergence of phage-insensitive or phage-resistant strains, as suggested by Guo et al. (2019), may also explain the reduced efficacy observed in the present study [50]. 

In summary, the heterogeneous phage activity observed among the *P. aeruginosa* planktonic cells may be related to differences in phage–host interactions, including variability in phage receptors, adsorption efficiency, intracellular replication, and bacterial defense mechanisms [52]. These differences may also explain the distinct responses observed between JG005 and JG024, including isolates susceptible to only one of the two phages. 

### 4.2. Antibiofilm Ability of Phages JG005 and JG024

Biofilm formation represents a crucial virulence factor in *P. aeruginosa*, frequently contributing to antibiotic treatment failure. Biofilm-forming strains are usually more resistant to antibiotics than their planktonic counterparts [40]. Therefore, understanding the prevalence of these strains is vital for managing *P. aeruginosa* infections in veterinary medicine. In this study, 41.38% of the isolates were able to form biofilms, which is in accordance with Pye et al. (2013) [53], and slightly lower than the higher biofilm-forming capacity reported by Płókarz et al. [40].

Bacteriophages have shown promising results in degrading and inactivating biofilms and are considered a potential alternative or adjunctive treatment for biofilm-related infections in veterinary settings. In this study, only one isolate showed strong biofilm reduction after a four-hour exposure to JG005 and JG024, which is consistent with the findings presented by Guo et al. [50]. These authors reported a significant recovery of metabolic activity after 24 h of treatment. This may explain why, in the present study, no isolate showed substantial (≥50%) inhibition at 24 h of treatment with either phage. Furthermore, JG024 was associated with a negative percentage of biofilm reduction, with biofilm regrowth, indicating poor biofilm-disrupting capacity. This finding is in accordance with Magin et al. (2019), who also reported that JG024 was less effective against biofilms [54]. This reduced phage activity against biofilms may be associated with restricted diffusion through the extracellular polymeric substance matrix and the presence of phenotypically heterogeneous bacterial subpopulations, which together limit phage access to target cells within the biofilm structure [55]. Although the efficacy of single phages against planktonic cells was demonstrated, their performance against biofilm-producing isolates was less effective. The use of a bacteriophage cocktail is therefore suggested as a future strategy to address this limitation, given the demonstrated ability of phage cocktails to infiltrate and disrupt *P. aeruginosa* biofilms [19,56,57]. 

Although bacteriophages offer an alternative to antibiotics for the control of MDR infections, some bacteria are developing resistance to phages. The coevolution of phages and bacteria is a reciprocal process of adaptation and counter-adaptation that plays a crucial role in shaping microbial communities [58,59]. In the present study, the bacterial regrowth observed in some TKAs after 24h may suggest the emergence or selection of phage-insensitive or phage-resistant bacterial subpopulations during prolonged phage exposure [58]. The heterogeneous response observed among isolates may also be related to differences in phage–host interactions, including receptor availability, adsorption efficiency, and bacterial defense mechanisms [45,58]. As previously suggested, future phage interventions may use cocktails targeting multiple receptors to mitigate resistance [45]. It is important to note that, in this study, the relatively short duration of the experimental assays may not allow us to fully capture the potential for the development of bacteriophage resistance over prolonged exposure. As bacterial resistance to phages can emerge through rapid adaptive mechanisms [58], the observed efficacy may not necessarily reflect long-term therapeutic outcomes. 

While the present study focused on JG005 and JG024, other effective phages against *P. aeruginosa* isolates from veterinary sources have been identified [47,48,56]. The present study complements the previous work by Lourenço et al. (2025) by evaluating JG005 and JG024 in a distinct collection of companion-animal *P. aeruginosa* isolates and using 24 h TKAs at different input MOIs. These findings provide additional insights into the kinetics and variability of phage activity. Further work should focus on the evaluation of different phages, the isolation of new ones, and the study of phage cocktails to improve the antimicrobial ability of this innovative approach in veterinary clinical settings. The heterogeneous host range observed for JG005 and JG024 also highlights the importance of selecting phages according to the susceptibility profile of individual clinical isolates, particularly when considering their potential therapeutic application. In addition, combination therapy involving bacteriophages and antibiotics also represents a promising strategy for the treatment of MDR infections, improving therapeutic efficacy. This approach has been increasingly reported in both human and veterinary settings [50,51,52,53,54,55,56,57,58,59,60,61,62]. Furthermore, phage–antibiotic synergy may enhance bacterial clearance while potentially reducing the likelihood of resistance development [61].

It should also be noted that the number of *P. aeruginosa* isolates included in this study (n = 29) may limit the generalization of the findings. Furthermore, whole-genome sequencing was not performed in the present study, as the primary focus was the phenotypic characterization of antimicrobial resistance and the evaluation of phage susceptibility and activity. Genomic characterization of these isolates in future studies would provide further insights into the mechanisms underlying phage susceptibility, resistance, and bacterial adaptation. Moreover, although the Congo Red agar method used to assess biofilm production has been successfully applied in previous studies [16,63], its qualitative nature and reported variability compared with microplate-based assays should be considered when interpreting the biofilm production results. Finally, although antibiofilm activity was assessed using an AB assay, this method primarily reflects metabolic activity, which was evaluated at a single time point. As such, it does not directly demonstrate structural disruption or eradication of the biofilm. Future studies incorporating complementary biofilm assessment methods, colony counting approaches and more detailed kinetic analyses would provide a more comprehensive understanding of bacteriophage efficacy.

## 5. Conclusions

AMR is responsible for the increase in difficult-to-treat infections, raising concerns about the future of healthcare. *P. aeruginosa*, frequently characterized by an MDR profile and biofilm-forming ability, can cause several complicated infections in humans and animals. In this study, the antimicrobial activity of the bacteriophages JG005 and JG024 was evaluated against a collection of *P. aeruginosa* isolates, obtained from clinical samples of companion animals. The results revealed that most of the isolates were susceptible to both bacteriophages. TKA showed that JG005 inhibited the growth of 94.12% (n = 16/17) of the studied isolates at all tested MOIs and JG024 inhibited 94.74% (n = 18/19) of the isolates. Considering biofilm cells, JG005 showed strong antimicrobial suppression of one isolate after four hours at all MOIs and JG024 showed strong antimicrobial suppression of one isolate after four hours at MOI 100.

This study underscores the potential of bacteriophages as an alternative or complementary therapy against antibiotic-resistant *P. aeruginosa* infections, highlighting the need for further research to optimize their use against biofilms. Future studies should evaluate additional phages, phage combinations and complementary approaches, before the therapeutic applicability of these phages in veterinary clinical settings can be established.

## Figures and Tables

**Table 1:** Resistance percentage and number of antibiotic-resistant (R), intermediate (I) and susceptible (S) *P. aeruginosa* isolates obtained from dogs and cats.

Antibiotic	Isolates Tested (n)	Resistance (%)	S (n)			R (n)			I (n)		
			Dogs	Cats	Total	Dogs	Cats	Total	Dogs	Cats	Total
Amikacin	26	0.00	19	7	26	0	0	0	0	0	0
Tobramycin	26	0.00	21	5	26	0	0	0	0	0	0
Gentamicin	24	4.16	14	6	20	0	1	1	2	1	3
Streptomycin	7	85.71	0	-	0	6	-	6	1	-	1
Carbenicillin	22	68.18	7	0	7	9	6	15	0	0	0
Piperacillin	27	3.70	20	6	26	1	0	1	0	0	0
Cephalexin	26	88.46	3	0	3	15	8	23	0	0	0
Cefoperazone	4	25.00	2	0	2	0	1	1	1	0	1
Ceftazidime	27	14.81	19	4	23	2	2	4	0	0	0
Cefovecin	2	100	0	-	0	2	-	2	0	-	0
Ciprofloxacin	29	13.79	20	5	25	1	3	4	0	0	0
Enrofloxacin	29	44.83	3	0	3	6	7	13	12	1	13
Ofloxacin	27	14.81	19	1	20	1	3	4	1	2	3
Marbofloxacin	22	36.36	12	1	13	1	7	8	1	0	1
Minocycline	1	100	0	-	0	1	-	1	0	-	0
Doxycycline	1	100	0	-	0	1	-	1	0	-	0
Nitrofurantoin	6	100	0	0	0	3	3	6	0	0	0

Legend: n—number of isolates. %—percentage. A hyphen (-) means that the antibiotic was not tested.

**Table 2:** Susceptibility of the bacterial collection to bacteriophages JG005 and JG024 based on spot-test results.

Isolate ID	Bacteriophage JG005	Bacteriophage JG024
**16/19-1**	susceptible	susceptible
**16/19-2**	susceptible	susceptible
**82/19-1 B**	susceptible	susceptible
**625/19-1 A**	susceptible	susceptible
**625/19-1 (2)1**	NS	susceptible
**626/19-1 2(1)**	NS	NS
**626/19-1 3(1)**	NS	susceptible
**638/19-1 1**	susceptible	susceptible
**670/ 19-1 (2) 1**	susceptible	susceptible
**163/20-1 I**	NS	susceptible
**219/20-1 1(1)**	NS	NS
**963/20-1 (2) 1**	NS	NS
**1047/20-1 1**	susceptible	susceptible
**1086/20-1 1**	susceptible	susceptible
**499/21-2 1(1)**	NS	NS
**553/21-1**	NS	NS
**588/21-1 1**	susceptible	susceptible
**604/21-1 1**	susceptible	NS
**616/21-(1) +(1)**	susceptible	susceptible
**616/21-(2) 1**	susceptible	susceptible
**645/21-1**	susceptible	susceptible
**743/21-1**	susceptible	susceptible
**784/21-1**	NS	NS
**864/21-2 1**	NS	NS
**981/21-1**	susceptible	susceptible
**1082/22-1**	NS	NS
**1162/22-1**	NS	susceptible
**1240/22-1**	susceptible	NS
**1289/22-1**	susceptible	susceptible
**Total**	**17**	**19**

Legend: NS—non-susceptible: ID—identification.

**Table 3:** Mean TKA results for bacteriophage JG005 after 24h incubation.

Isolate	Positive Control		MOI 1		MOI 10		MOI 100	
	µ	SD	µ	SD	µ	SD	µ	SD
16/19-1	1.58	0.06	0.08	0.17	0.17	0.15	0.03	0.06
16/19-2	1.66	0.09	0.09	0.06	0.13	0.09	0.04	0.04
82/19-1 B	0.54	0.50	0.43	0.16	0.44	0.15	0.17	0.07
625/19-1 A	1.44	0.19	0.58	0.20	0.72	0.24	0.91	0.34
638/19-1 1	1.15	0.56	0.38	0.25	0.50	0.34	0.23	0.31
670/ 19-1 (2) 1	1.59	0.11	0.24	0.03	0.29	0.03	0.18	0.08
1047/20-1 1	1.40	0.52	1.07	0.14	1.06	0.20	0.63	0.42
1086/20-1 1	1.01	0.50	1.07	0.14	1.17	0.18	0.11	0.31
588/21-1 1	0.86	0.55	0.79	0.07	0.51	0.19	0.01	0.06
604/21-1 1	1.21	0.29	0.60	0.29	0.59	0.26	0.38	0.22
616/21-(1) +(1)	0.73	0.34	0.13	0.14	0.24	0.08	0.12	0.15
616/21-(2) 1	0.88	0.08	0.47	0.15	0.47	0.07	0.28	0.11
645/21-1	1.53	0.46	0.11	0.22	0.24	0.10	0.13	0.01
743/21-1	1.37	0.09	0.69	0.31	1.02	0.15	1.04	0.08
981/21-1	0.25	0.00	0.06	0.06	0.09	0.01	0.08	0.07
1240/22-1	1.73	0.07	1.14	0.24	1.22	0.17	1.23	0.26
1289/22-1	1.45	0.20	1.10	0.06	1.15	0.04	1.00	0.28
Reference strain DSM 19880	1.65	0.32	0.15	0.16	0.19	0.20	0.42	0.36

Legend: Values are expressed as mean OD600 values ± SD across three independent assays. µ—mean; SD—standard deviation. MOI—multiplicity of infection.

**Table 4:** Mean TKA results for bacteriophage JG024 at the 24h timepoint.

Isolate	Positive Control		MOI 1		MOI 10		MOI 100	
	µ	SD	µ	SD	µ	SD	µ	SD
16/19-1	1.91	0.18	0.00	0.47	0.55	0.53	0.04	0.07
16/19-2	1.59	0.34	0.47	0.32	0.56	0.26	0.29	0.20
82/19-1 B	0.81	0.15	0.11	0.12	0.16	0.24	0.10	0.13
625/19-1 A	0.94	0.48	0.58	0.31	0.61	0.36	0.59	0.44
625/19-1 (2)1	1.20	0.23	1.12	0.20	1.11	0.28	0.64	0.66
626/19-1 3(1)	1.39	0.26	0.91	0.22	1.04	0.34	1.53	0.13
638/19-1 1	1.57	0.13	1.07	0.08	0.96	0.22	1.15	0.13
670/ 19-1 (2) 1	1.37	0.55	0.31	0.14	0.44	0.10	0.35	0.01
163/20-1 I	1.56	0.52	0.93	0.22	0.80	0.34	0.93	0.32
1047/20-1 1	1.51	0.54	0.07	0.19	0.43	0.28	0.56	0.22
1086/20-1 1	1.71	0.03	0.83	0.79	1.00	0.59	0.62	0.47
588/21-1 1	1.16	0.12	0.37	0.29	0.77	0.35	0.44	0.52
616/21-(1) +(1)	0.52	0.08	0.49	0.26	0.38	0.05	0.21	0.18
616/21-(2) 1	0.92	0.26	0.31	0.25	0.26	0.38	0.22	0.03
645/21-1	1.51	0.37	0.06	0.31	0.05	0.40	0.02	0.03
743/21-1	1.21	0.03	0.39	0.27	0.06	0.26	0.33	0.19
981/21-1	0.23	0.18	0.05	0.26	0.16	0.37	0.08	0.07
1162/22-1	1.89	0.09	0.64	0.09	0.99	0.38	1.63	0.40
1289/22-1	1.27	0.35	0.73	0.20	0.68	0.57	1.09	0.43
Reference DSM 19882	0.62	0.25	0.00	0.25	0.13	0.30	0.12	0.24

Legend: Values are expressed as mean OD600 values ± SD across three independent assays. µ—mean; SD—standard deviation. MOI—multiplicity of infection.

**Table 5:** Statistical analysis of bacterial growth following JG005 exposure at MOIs 1, 10, and 100 compared with the positive control after 24 h.

Comparison	Estimated Difference in OD600 (MOI − Control)	95% CI	Adjusted *p*-Value
MOI 1 vs. Control	−0.729	[−0.879, −0.578]	<0.0001
MOI 10 vs. Control	−0.667	[−0.818, −0.517]	<0.0001
MOI 100 vs. Control	−0.844	[−0.994, −0.693]	<0.0001

Legend: Estimated differences in OD600 were obtained from a linear mixed-effects model, with the treatment group as a fixed effect and the isolate as a random intercept. Negative estimates indicate lower OD600 values in phage-treated groups than in the positive control. Post hoc comparisons of each multiplicity of infection (MOI) with the positive control were performed using Dunnett’s test. Values are presented as estimated differences with 95% confidence intervals (CI). Adjusted *p*-values ≤ 0.05 were considered statistically significant.

**Table 6:** Statistical analysis of bacterial growth following JG024 exposure at MOIs 1, 10, and 100 compared with the positive control after 24 h.

Comparison	Estimated Difference in OD600 (MOI − Control)	95% CI	Adjusted *p*-Value
MOI 1 vs. Control	−0.778	[−0.938, −0.617]	<0.0001
MOI 10 vs. Control	−0.688	[−0.849, −0.528]	<0.0001
MOI 100 vs. Control	−0.687	[−0.848, −0.526]	<0.0001

Legend: Estimated differences in OD600 were obtained from a linear mixed-effects model, with the treatment group as a fixed effect and the isolate as a random intercept. Negative estimates indicate lower OD600 values in phage-treated groups than in the positive control. Post hoc comparisons of each multiplicity of infection (MOI) with the positive control were performed using Dunnett’s test. Values are presented as estimated differences with 95% confidence intervals (CI). Adjusted *p*-values ≤ 0.05 were considered statistically significant.

**Table 7:** Statistical analysis of pairwise comparisons between JG005 MOIs (1, 10, and 100) at each time point using Tukey’s test.

Comparison	Estimated Difference (% Biofilm Reduction)	95% CI	Adjusted *p*-Value
MOI 1 vs. MOI 10 (4 h)	3.10	[−19.3, 25.5]	0.9413
MOI 1 vs. MOI 100 (4 h)	4.33	[−18.1, 26.8]	0.8887
MOI 10 vs. MOI 100 (4 h)	1.23	[−21.2, 23.7]	0.9905
MOI 1 vs. MOI 10 (24 h)	2.58	[−19.8, 25.0]	0.9589
MOI 1 vs. MOI 100 (24 h)	2.83	[−19.6, 25.2]	0.9509
MOI 10 vs. MOI 100 (24 h)	0.25	[−22.2, 22.7]	0.9996

Legend: MOI—multiplicity of infection; CI—confidence interval.

**Table 8:** Statistical analysis of comparisons between 4 h and 24 h exposure at each MOI for the JG005 biofilm assay using Holm’s method.

Comparison	Estimated Difference (% Biofilm Reduction)	95% CI	Adjusted *p*-Value
4 h vs. 24 h (MOI 1)	9.61	[−9.06, 28.3]	0.3075
4 h vs. 24 h (MOI 10)	9.09	[−9.58, 27.8]	0.3341
4 h vs. 24 h (MOI 100)	8.11	[−10.56, 26.8]	0.3886

Legend: MOI—multiplicity of infection; CI—confidence interval.

**Table 9:** Statistical analysis of pairwise comparisons between JG024 MOIs (1, 10, and 100) at each time point using Tukey’s test.

Comparison	Estimated Difference (% Biofilm Reduction)	95% CI	Adjusted *p*-Value
MOI 1 vs. MOI 10 (4 h)	15.98	[−2.71, 34.67]	0.1096
MOI 1 vs. MOI 100 (4 h)	−8.36	[−27.05, 10.33]	0.5397
MOI 10 vs. MOI 100 (4 h)	−24.34	[−43.03, −5.65]	0.0070
MOI 1 vs. MOI 10 (24 h)	4.27	[−14.41, 22.96]	0.8503
MOI 1 vs. MOI 100 (24 h)	−0.41	[−19.09, 18.28]	0.9985
MOI 10 vs. MOI 100 (24 h)	−4.68	[−23.37, 14.01]	0.8234

Legend: MOI—multiplicity of infection; CI—confidence interval.

**Table 10:** Statistical analysis of comparisons between 4 h and 24 h exposure at each MOI for the JG024 biofilm assay using Holm’s method.

Comparison	Estimated Difference (% Biofilm Reduction)	95% CI	Adjusted *p*-Value
4 h vs. 24 h (MOI 1)	−10.88	[−26.5, 4.71]	0.1696
4 h vs. 24 h (MOI 10)	−22.59	[−38.2, −7.00]	0.0049
4 h vs. 24 h (MOI 100)	−2.93	[−18.5, 12.66]	0.7108

Legend: MOI—multiplicity of infection; CI—confidence interval.

## Data Availability

The original contributions presented in this study are included in the article/Supplementary Materials. Further inquiries can be directed to the corresponding authors.

## Supplementary Materials

The following supporting information can be downloaded at: https://www.mdpi.com/article/10.3390/pathogens15101067/s1, Supplementary File S1: Detailed antimicrobial susceptibility testing results for the 29 *P. aeruginosa* isolates; Supplementary File S2: Graphic representation of all time kill assays performed to each susceptible *P. aeruginosa* isolate using bacteriophage JG005 and JG024.; Supplementary File S3: Percentage of area under the curve (AUC) inhibition for *P. aeruginosa* isolates following bacteriophage exposure at MOI 1, 10, and 100.
