# Supplementary material for: Targeting Multidrug-Resistant Pseudomonas aeruginosa from Companion Animals Using Bacteriophages
Source: Pathogens. 2026 Oct 8;15(10):1067. doi: 10.3390/pathogens15101067 (PMC13708516; doi:10.3390/pathogens15101067)
Supplement: Supplementary file 1 [file pathogens-15-01067-s001.zip › Supplementary file S2.pdf]

Supplementary file S2: Graphic representation of all time kill assays performed to each susceptible *P. aeruginosa* isolate and respective bacteriophage JG005 and JG024.

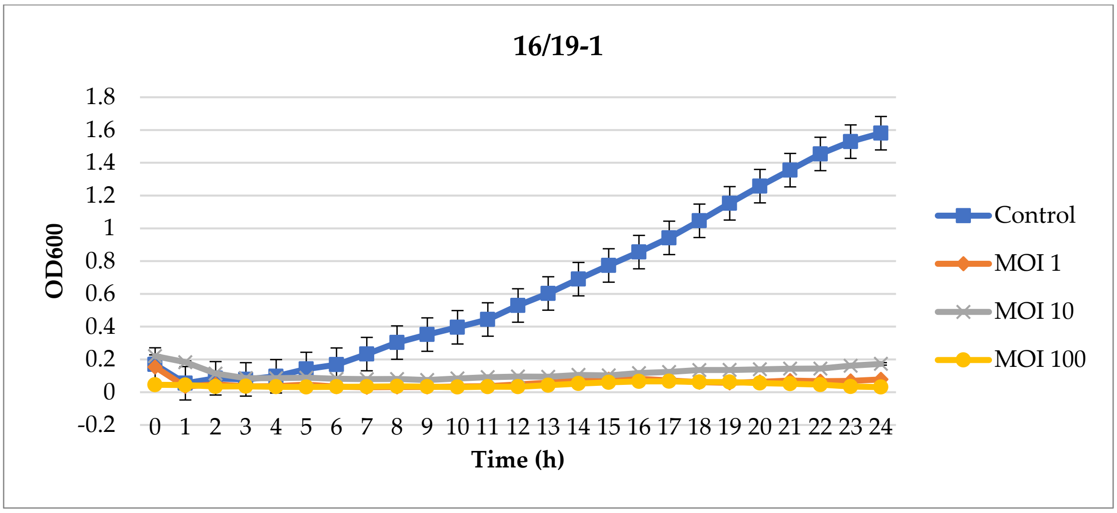

Figure S1- Time-kill assay of bacteriophage JG005 against *P. aeruginosa* 16/19-1 isolate. The bacterial culture was infected with phage JG005 at MOI 1 (orange, ♦), MOI 10 (gray, ×), and MOI 100 (yellow, ●). The bacterial culture not treated with bacteriophage (blue, ■) was used as a positive control. Optical density at 600 nm was measured hourly for 24 hours. Each data point represents the mean of 3 assays, each one with 3 replicas (n=9). Error bars represent standard errors based on the control series.

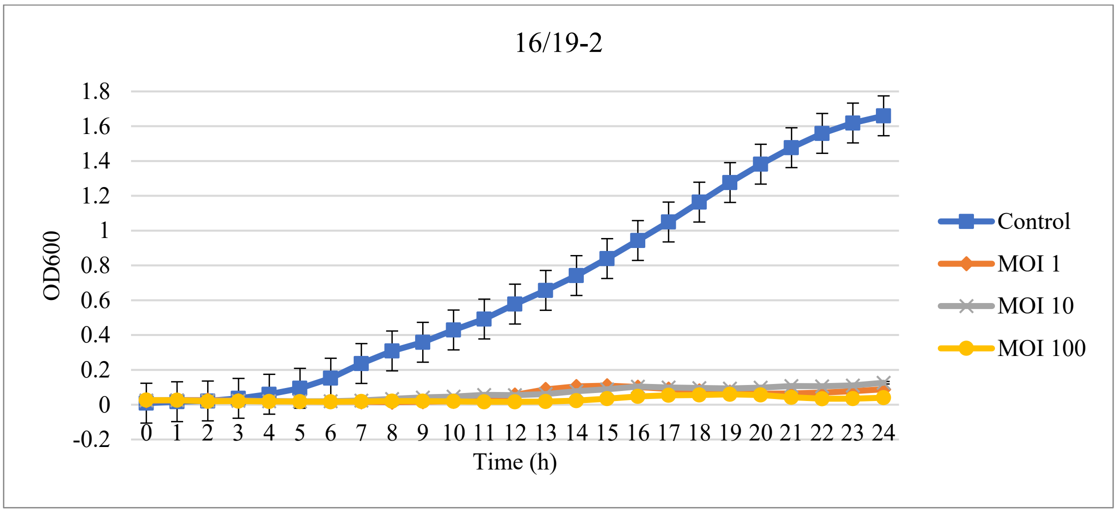

Figure S2- Time-kill assay of bacteriophage JG005 against *P. aeruginosa* 16/19-2 isolate. The bacterial culture was infected with phage JG005 at MOI 1 (orange, ♦), MOI 10 (gray, ×), and MOI 100 (yellow, ●). The bacterial culture not treated with bacteriophage (blue, ■) was used as a positive control. Optical density at 600 nm was measured hourly for 24 hours. Each data point represents the mean of 3 assays, each one with 3 replicas (n=9). Error bars represent standard errors based on the control series.

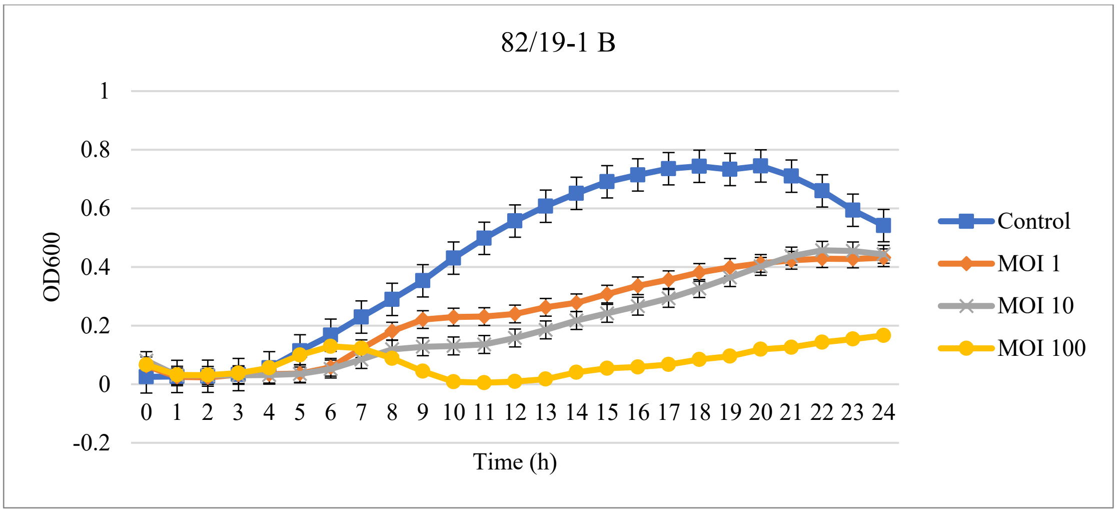

Figure S3- Time-kill assay of bacteriophage JG005 against *P. aeruginosa* 82/19-1 B isolate. The bacterial culture was infected with phage JG005 at MOI 1 (orange, ♦), MOI 10 (gray, ×), and MOI 100 (yellow, ●). The bacterial culture not treated with bacteriophage (blue, ■) was used as a positive control. Optical density at 600 nm was measured hourly for 24 hours. Each data point represents the mean of 3 assays, each one with 3 replicas (n=9). Error bars represent standard errors based on the control series.

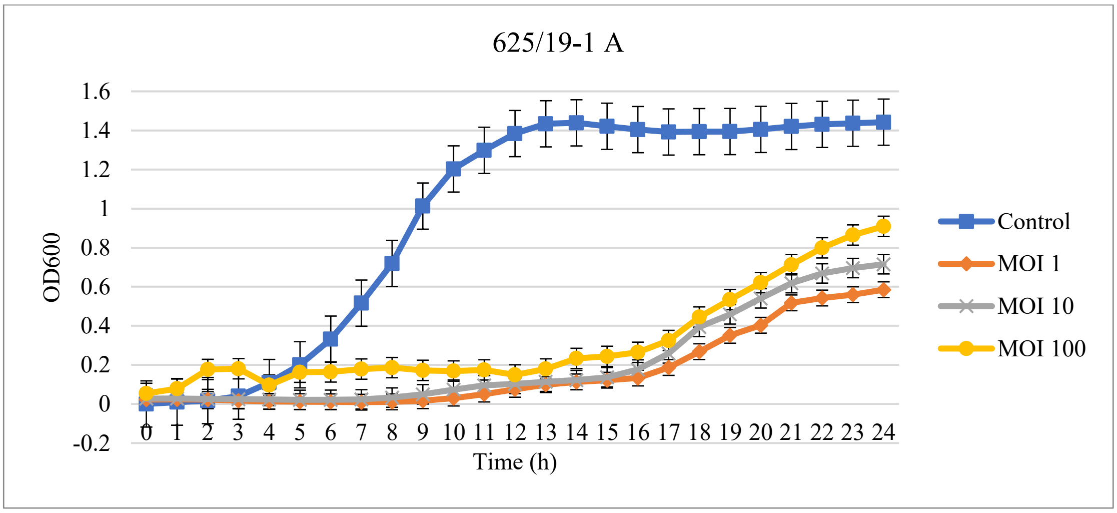

Figure S4- Time-kill assay of bacteriophage JG005 against *P. aeruginosa* 625/19-1 A isolate. The bacterial culture was infected with phage JG005 at MOI 1 (orange, ♦), MOI 10 (gray, ×), and MOI 100 (yellow, ●). The bacterial culture not treated with bacteriophage (blue, ■) was used as a positive control. Optical density at 600 nm was measured hourly for 24 hours. Each data point represents the mean of 3 assays, each one with 3 replicas (n=9). Error bars represent standard errors based on the control series.

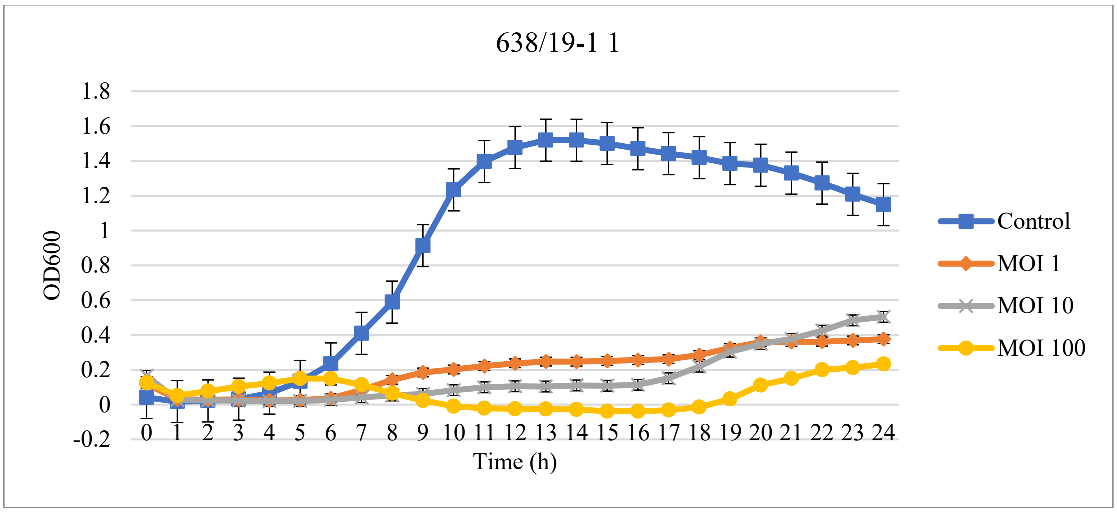

Figure S5- Time-kill assay of bacteriophage JG005 against *P. aeruginosa* 638/19-1 1 isolate. The bacterial culture was infected with phage JG005 at MOI 1 (orange, ♦), MOI 10 (gray, ×), and MOI 100 (yellow, ●). The bacterial culture not treated with bacteriophage (blue, ■) was used as a positive control. Optical density at 600 nm was measured hourly for 24 hours. Each data point represents the mean of 3 assays, each one with 3 replicas (n=9). Error bars represent standard errors based on the control series.

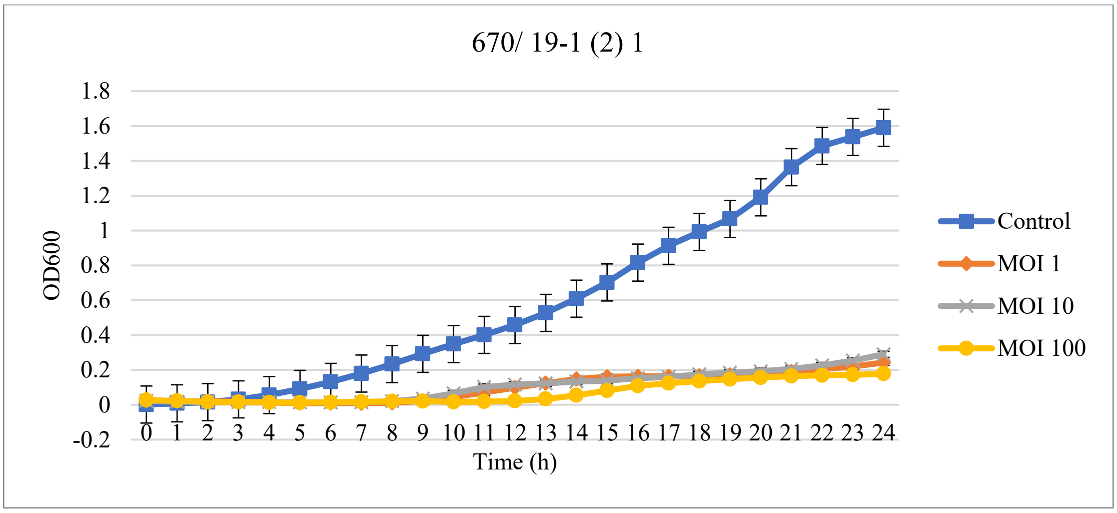

Figure S6- Time-kill assay of bacteriophage JG005 against *P. aeruginosa* 670/19-1 (2) 1 isolate. The bacterial culture was infected with phage JG005 at MOI 1 (orange, ♦), MOI 10 (gray, ×), and MOI 100 (yellow, ●). The bacterial culture not treated with bacteriophage (blue, ■) was used as a positive control. Optical density at 600 nm was measured hourly for 24 hours. Each data point represents the mean of 3 assays, each one with 3 replicas (n=9). Error bars represent standard errors based on the control series.

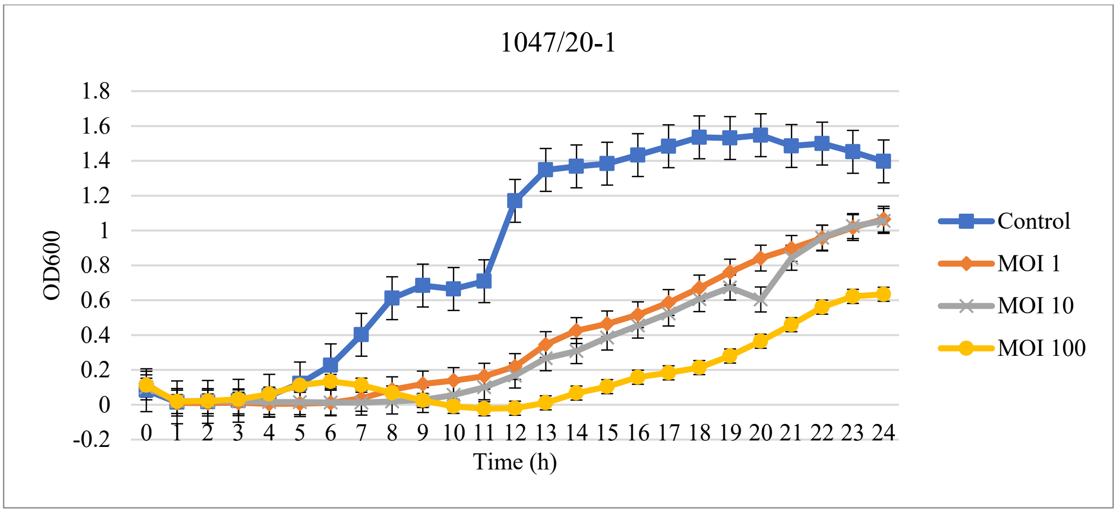

Figure S7- Time-kill assay of bacteriophage JG005 against *P. aeruginosa* 1047/20-1 isolate. The bacterial culture was infected with phage JG005 at MOI 1 (orange, ♦), MOI 10 (gray, ×), and MOI 100 (yellow, ●). The bacterial culture not treated with bacteriophage (blue, ■) was used as a positive control. Optical density at 600 nm was measured hourly for 24 hours. Each data point represents the mean of 3 assays, each one with 3 replicas (n=9). Error bars represent standard errors based on the control series.

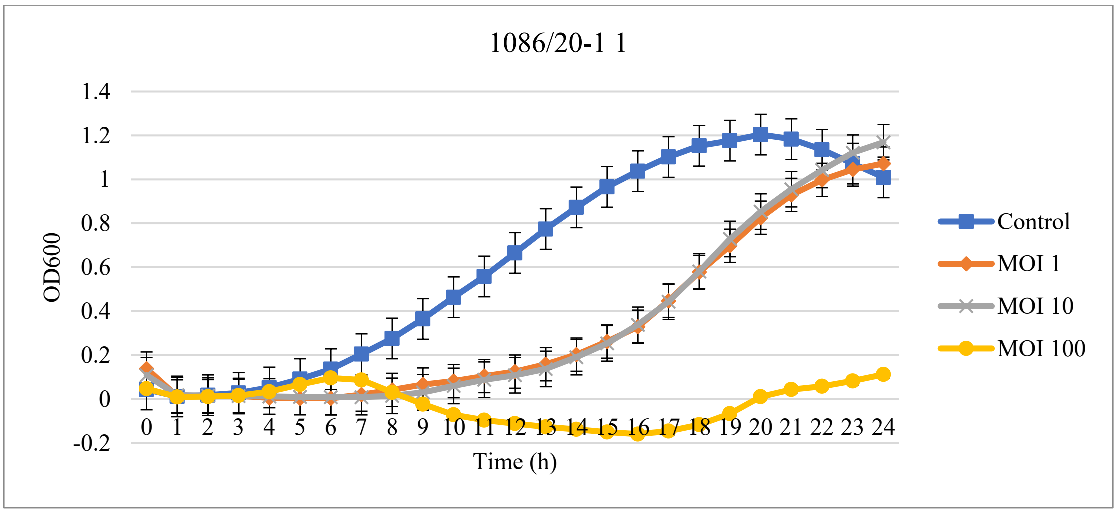

Figure S8- Time-kill assay of bacteriophage JG005 against *P. aeruginosa* 1086/20-1 1 isolate. The bacterial culture was infected with phage JG005 at MOI 1 (orange, ♦), MOI 10 (gray, ×), and MOI 100 (yellow, ●). The bacterial culture not treated with bacteriophage (blue, ■) was used as a positive control. Optical density at 600 nm was measured hourly for 24 hours. Each data point represents the mean of 3 assays, each one with 3 replicas (n=9). Error bars represent standard errors based on the control series.

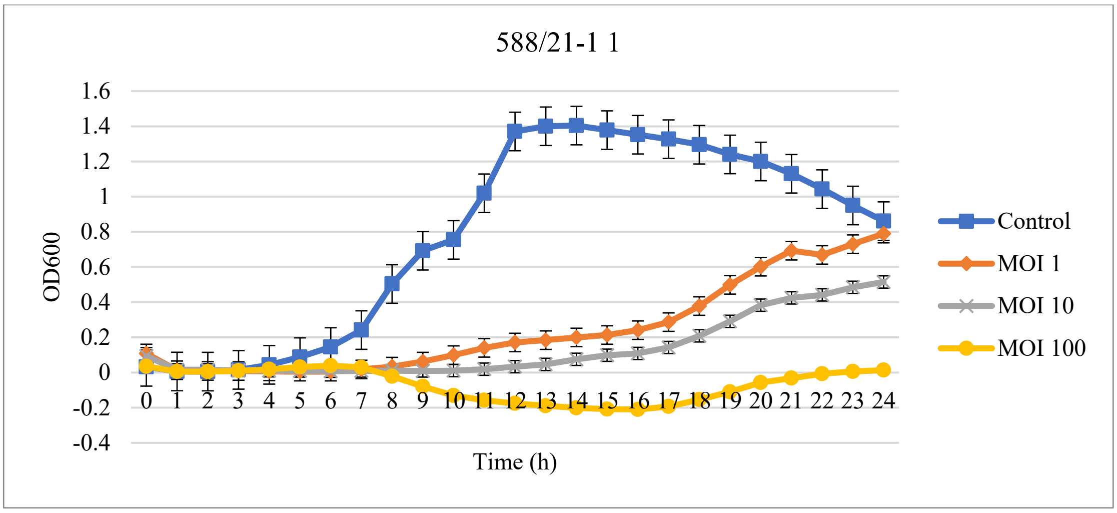

Figure S9- Time-kill assay of bacteriophage JG005 against *P. aeruginosa* 588/21-1 1 isolate. The bacterial culture was infected with phage JG005 at MOI 1 (orange, ♦), MOI 10 (gray, ×), and MOI 100 (yellow, ●). The bacterial culture not treated with bacteriophage (blue, ■) was used as a positive control. Optical density at 600 nm was measured hourly for 24 hours. Each data point represents the mean of 3 assays, each one with 3 replicas (n=9). Error bars represent standard errors based on the control series.

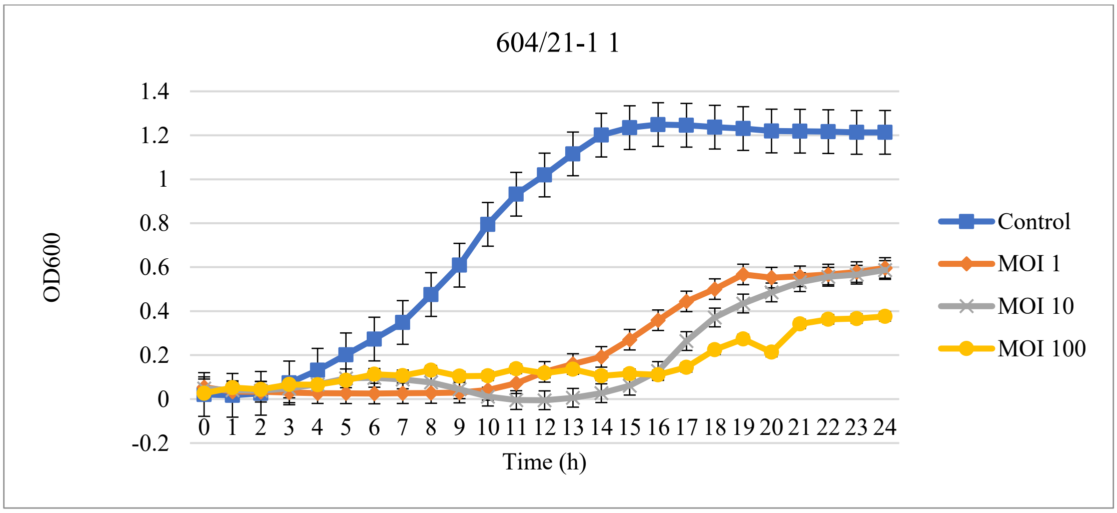

Figure S10- Time-kill assay of bacteriophage JG005 against *P. aeruginosa* 604/21-1 1 isolate. The bacterial culture was infected with phage JG005 at MOI 1 (orange, ♦), MOI 10 (gray, ×), and MOI 100 (yellow, ●). The bacterial culture not treated with bacteriophage (blue, ■) was used as a positive control. Optical density at 600 nm was measured hourly for 24 hours. Each data point represents the mean of 3 assays, each one with 3 replicas (n=9). Error bars represent standard errors based on the control series.

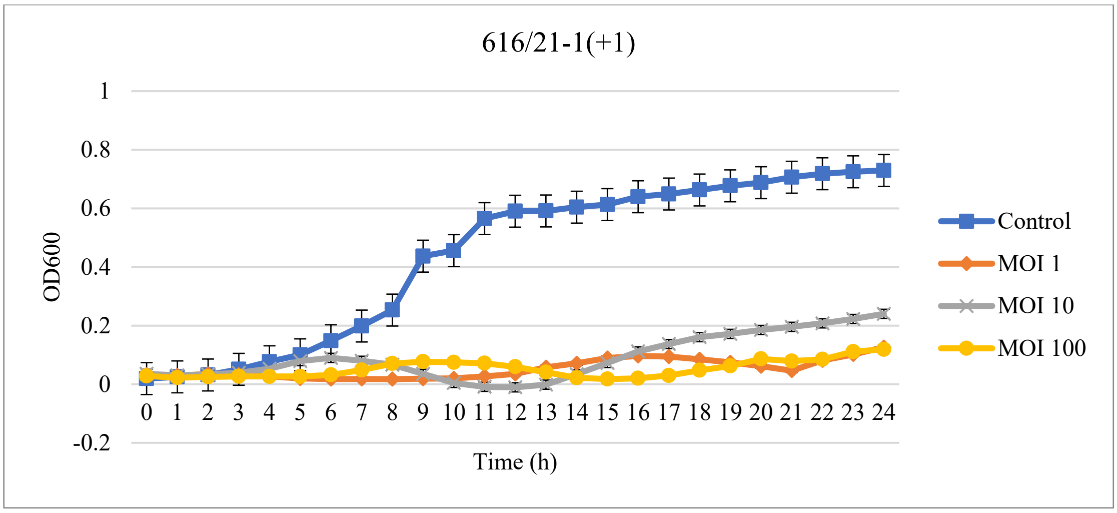

Figure S11- Time-kill assay of bacteriophage JG005 against *P. aeruginosa* 616/21-1(+1) isolate. The bacterial culture was infected with phage JG005 at MOI 1 (orange, ♦), MOI 10 (gray, ×), and MOI 100 (yellow, ●). The bacterial culture not treated with bacteriophage (blue, ■) was used as a positive control. Optical density at 600 nm was measured hourly for 24 hours. Each data point represents the mean of 3 assays, each one with 3 replicas (n=9). Error bars represent standard errors based on the control series.

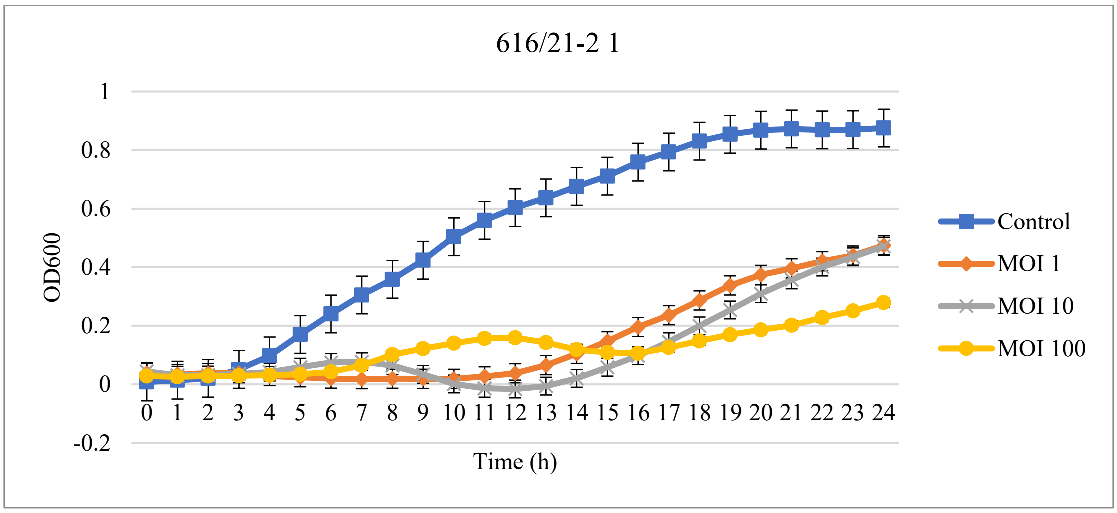

Figure S12- Time-kill assay of bacteriophage JG005 against *P. aeruginosa* 616/21-2 1 isolate. The bacterial culture was infected with phage JG005 at MOI 1 (orange, ♦), MOI 10 (gray, ×), and MOI 100 (yellow, ●). The bacterial culture not treated with bacteriophage (blue, ■) was used as a positive control. Optical density at 600 nm was measured hourly for 24 hours. Each data point represents the mean of 3 assays, each one with 3 replicas (n=9). Error bars represent standard errors based on the control series.

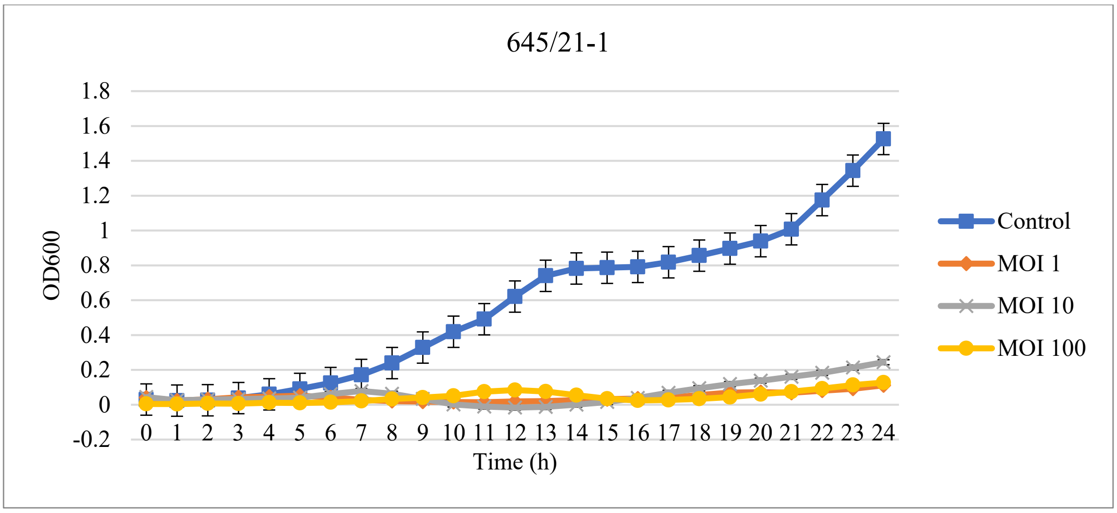

Figure S13- Time-kill assay of bacteriophage JG005 against *P. aeruginosa* 645/21-1 isolate. The bacterial culture was infected with phage JG005 at MOI 1 (orange, ♦), MOI 10 (gray, ×), and MOI 100 (yellow, ●). The bacterial culture not treated with bacteriophage (blue, ■) was used as a positive control. Optical density at 600 nm was measured hourly for 24 hours. Each data point represents the mean of 3 assays, each one with 3 replicas (n=9). Error bars represent standard errors based on the control series.

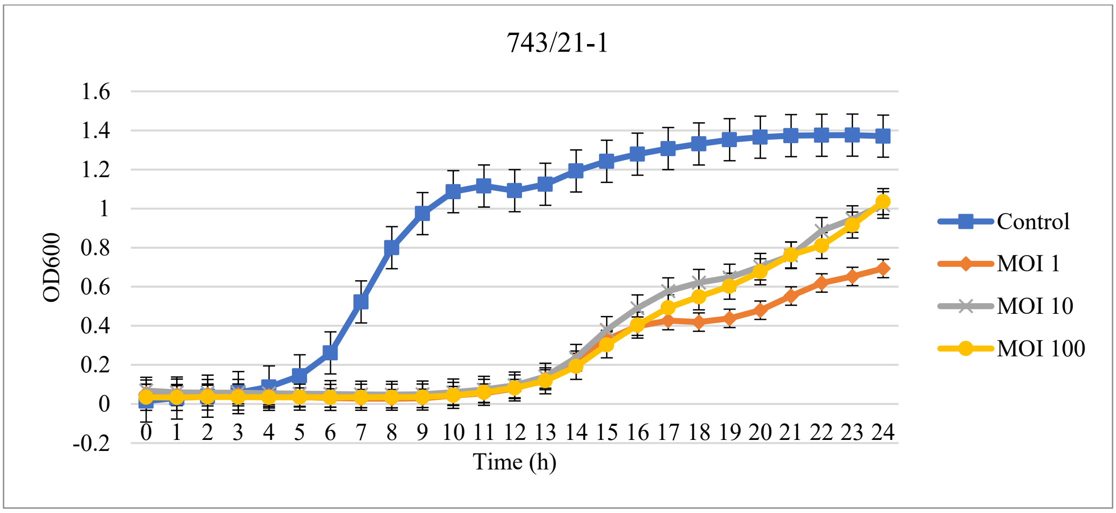

Figure S14- Time-kill assay of bacteriophage JG005 against *P. aeruginosa* 743/21-*1* isolate. The bacterial culture was infected with phage JG005 at MOI 1 (orange, ♦), MOI 10 (gray, ×), and MOI 100 (yellow, ●). The bacterial culture not treated with bacteriophage (blue, ■) was used as a positive control. Optical density at 600 nm was measured hourly for 24 hours. Each data point represents the mean of 3 assays, each one with 3 replicas (n=9). Error bars represent standard errors based on the control series.

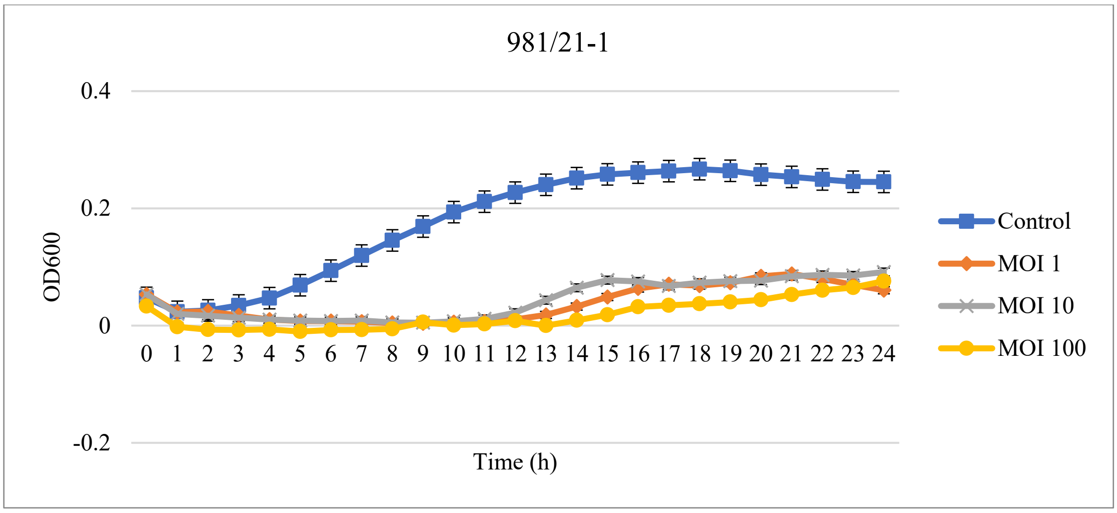

Figure S15- Time-kill assay of bacteriophage JG005 against *P. aeruginosa* 981/21-1 isolate. The bacterial culture was infected with phage JG005 at MOI 1 (orange, ♦), MOI 10 (gray, ×), and MOI 100 (yellow, ●). The bacterial culture not treated with bacteriophage (blue, ■) was used as a positive control. Optical density at 600 nm was measured hourly for 24 hours. Each data point represents the mean of 3 assays, each one with 3 replicas (n=9). Error bars represent standard errors based on the control series.

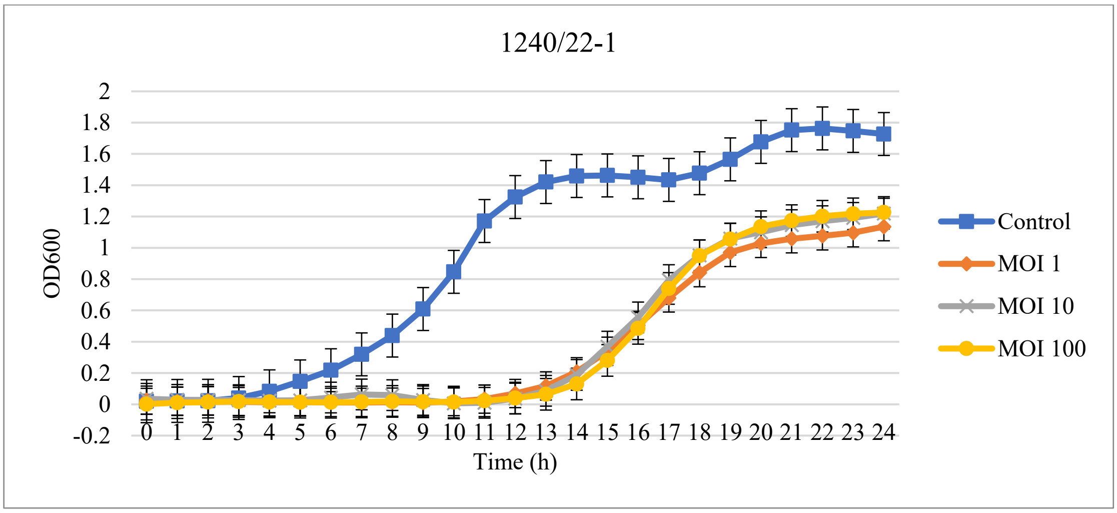

Figure S16 - Time-kill assay of bacteriophage JG005 against *P. aeruginosa* 1240/22-1 isolate. The bacterial culture was infected with phage JG005 at MOI 1 (orange, ♦), MOI 10 (gray, ×), and MOI 100 (yellow, ●). The bacterial culture not treated with bacteriophage (blue, ■) was used as a positive control. Optical density at 600 nm was measured hourly for 24 hours. Each data point represents the mean of 3 assays, each one with 3 replicas (n=9). Error bars represent standard errors based on the control series.

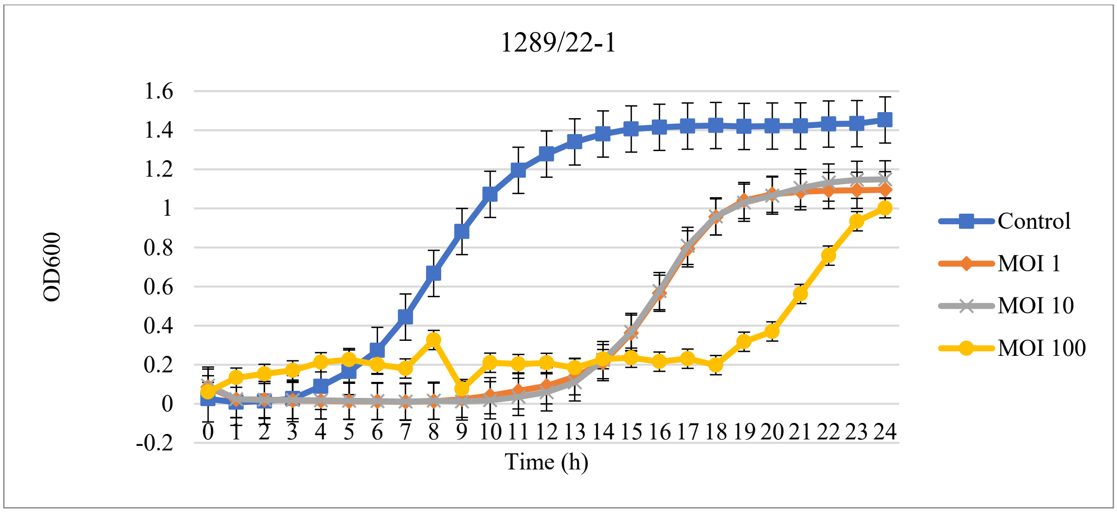

Figure S17- Time-kill assay of bacteriophage JG005 against *P. aeruginosa* 1289/22-1 isolate. The bacterial culture was infected with phage JG005 at MOI 1 (orange, ♦), MOI 10 (gray, ×), and MOI 100 (yellow, ●). The bacterial culture not treated with bacteriophage (blue, ■) was used as a positive control. Optical density at 600 nm was measured hourly for 24 hours. Each data point represents the mean of 3 assays, each one with 3 replicas (n=9). Error bars represent standard errors based on the control series.

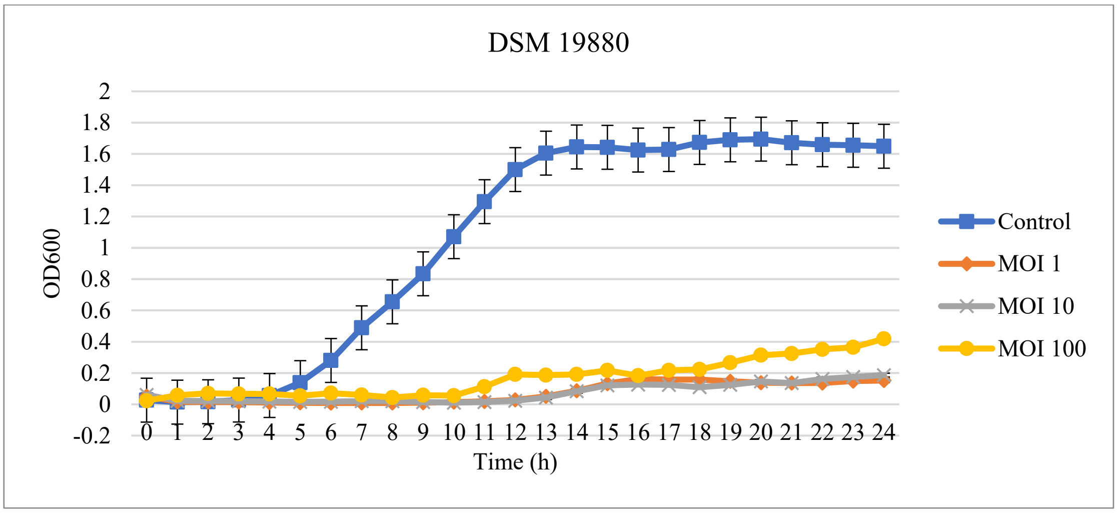

Figure S18- Time-kill assay of bacteriophage JG005 against *P. aeruginosa* DSM 19880. The bacterial culture was infected with phage JG005 at MOI 1 (orange, ♦), MOI 10 (gray, ×), and MOI 100 (yellow, ●). The bacterial culture not treated with bacteriophage (blue, ■) was used as a positive control. Optical density at 600 nm was measured hourly for 24 hours. Each data point represents the mean of 11 assays, each one with 3 replicas (n=33). Error bars represent standard errors based on the control series.

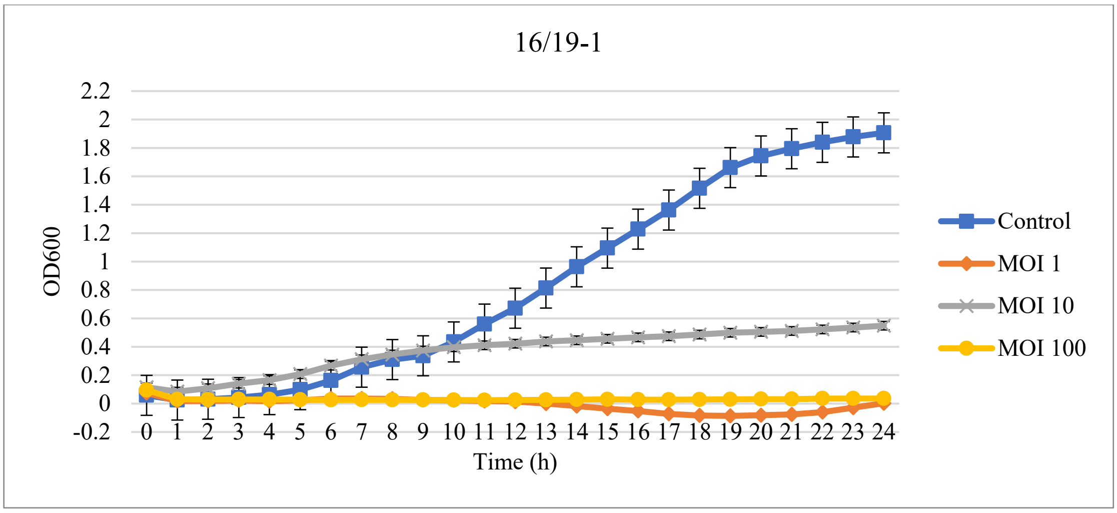

Figure S19- Time-kill assay of bacteriophage JG024 against *P. aeruginosa* 16/19-1 isolate. The bacterial culture was infected with phage JG024 at MOI 1 (orange, ♦), MOI 10 (gray, ×), and MOI 100 (yellow, ●). The bacterial culture not treated with bacteriophage (blue, ■) was used as a positive control. Optical density at 600 nm was measured hourly for 24 hours. Each data point represents the mean of 3 assays, each one with 3 replicas (n=9). Error bars represent standard errors based on the control series.

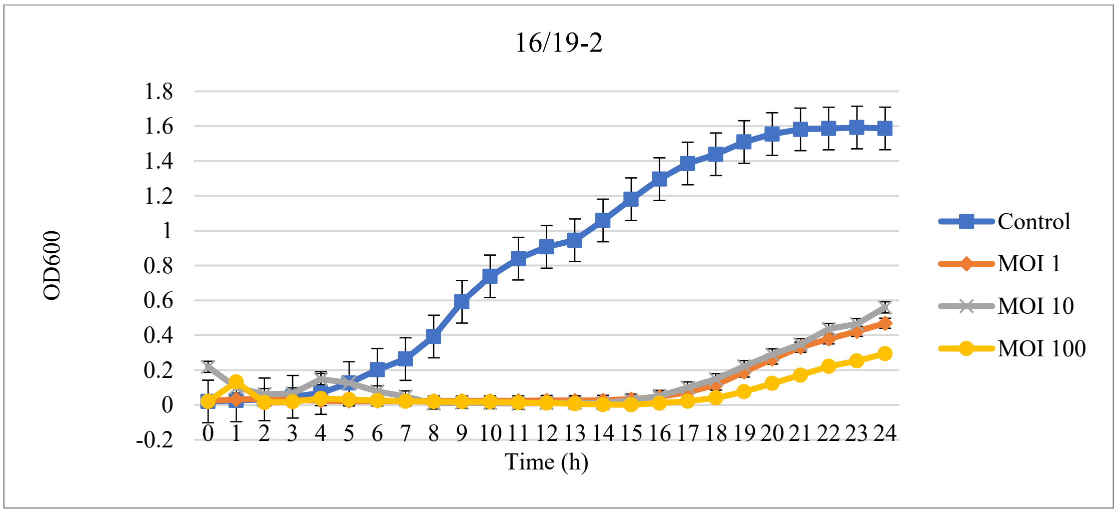

Figure S20- Time-kill assay of bacteriophage JG024 against *P. aeruginosa* 16/19-2 isolate. The bacterial culture was infected with phage JG024 at MOI 1 (orange, ♦), MOI 10 (gray, ×), and MOI 100 (yellow, ●). The bacterial culture not treated with bacteriophage (blue, ■) was used as a positive control. Optical density at 600 nm was measured hourly for 24 hours. Each data point represents the mean of 3 assays, each one with 3 replicas (n=9). Error bars represent standard errors based on the control series.

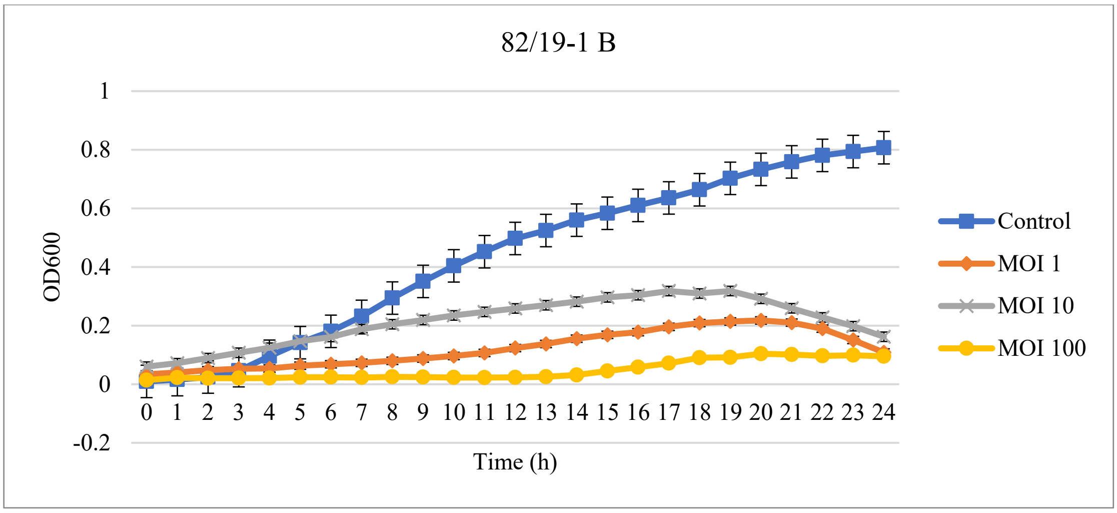

Figure S21- Time-kill assay of bacteriophage JG024 against *P. aeruginosa* 82/19-1 B isolate. The bacterial culture was infected with phage JG024 at MOI 1 (orange, ♦), MOI 10 (gray, ×), and MOI 100 (yellow, ●). The bacterial culture not treated with bacteriophage (blue, ■) was used as a positive control. Optical density at 600 nm was measured hourly for 24 hours. Each data point represents the mean of 3 assays, each one with 3 replicas (n=9). Error bars represent standard errors based on the control series.

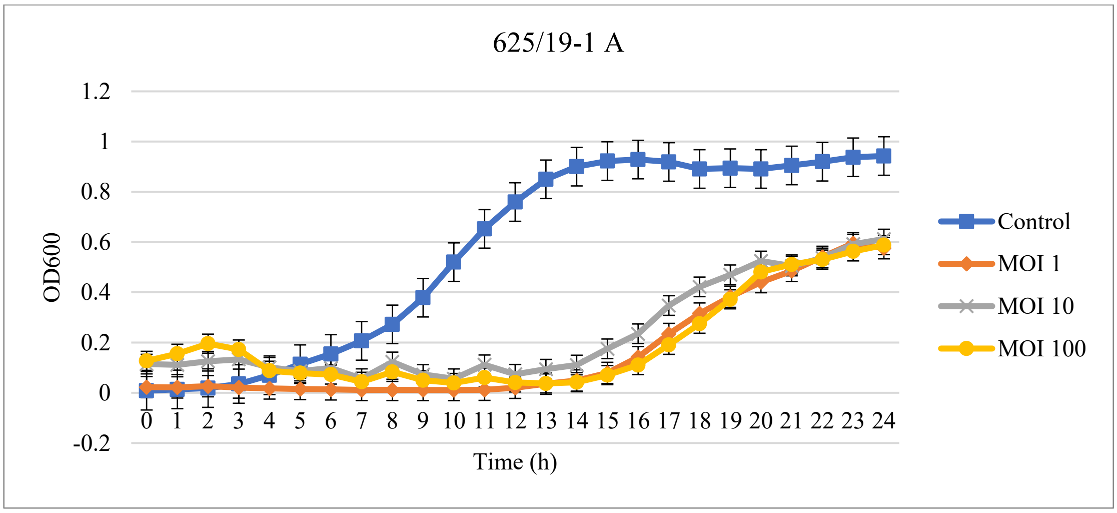

Figure S22 - Time-kill assay of bacteriophage JG024 against *P. aeruginosa* 625/19-1 A isolate. The bacterial culture was infected with phage JG024 at MOI 1 (orange, ♦), MOI 10 (gray, ×), and MOI 100 (yellow, ●). The bacterial culture not treated with bacteriophage (blue, ■) was used as a positive control. Optical density at 600 nm was measured hourly for 24 hours. Each data point represents the mean of 3 assays, each one with 3 replicas (n=9). Error bars represent standard errors based on the control series.

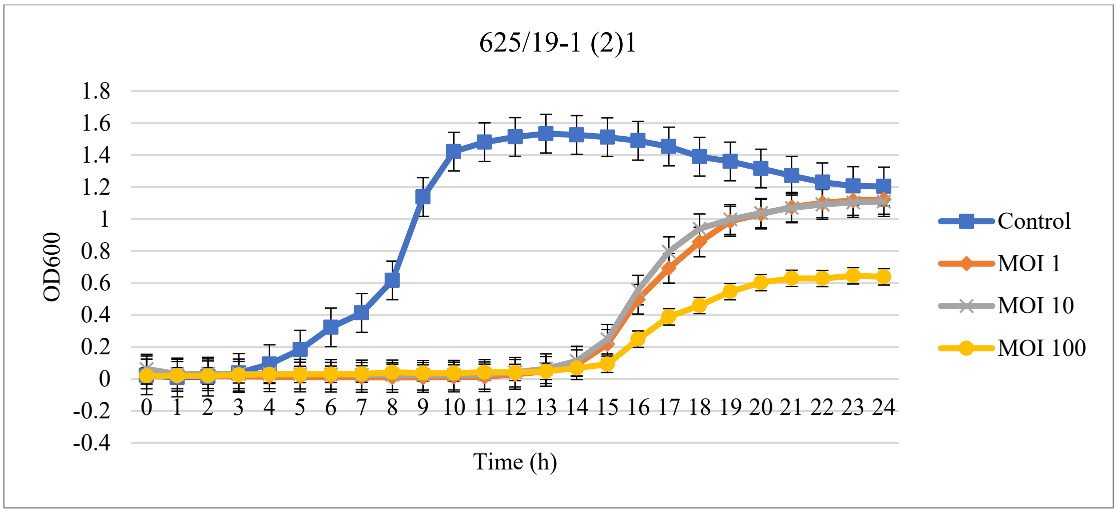

Figure S23- Time-kill assay of bacteriophage JG024 against *P. aeruginosa* 625/19-1 (2)1 isolate. The bacterial culture was infected with phage JG024 at MOI 1 (orange, ♦), MOI 10 (gray, ×), and MOI 100 (yellow, ●). The bacterial culture not treated with bacteriophage (blue, ■) was used as a positive control. Optical density at 600 nm was measured hourly for 24 hours. Each data point represents the mean of 3 assays, each one with 3 replicas (n=9). Error bars represent standard errors based on the control series.

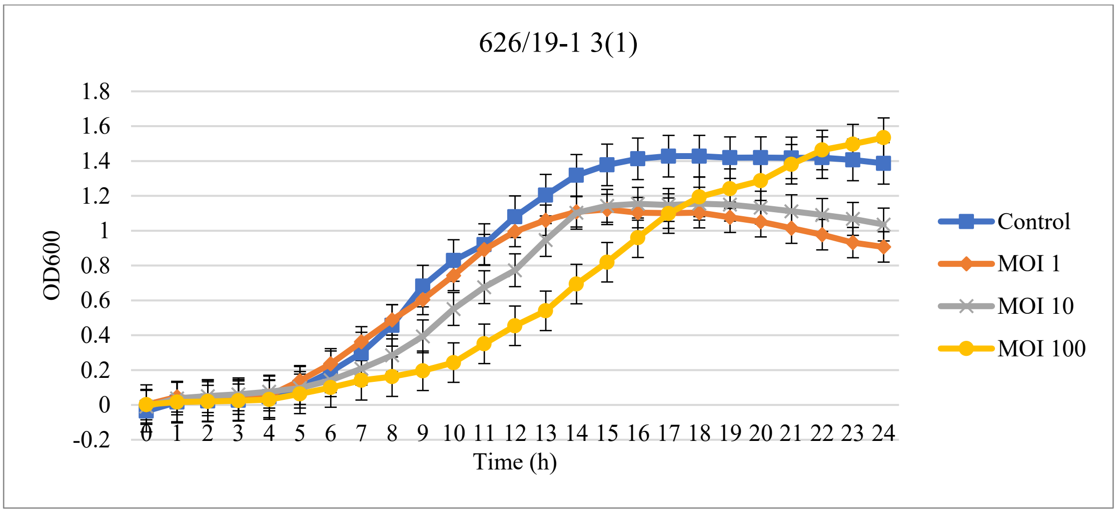

Figure S24- Time-kill assay of bacteriophage JG024 against *P. aeruginosa* 626/19-1 3(1) isolate. The bacterial culture was infected with phage JG024 at MOI 1 (orange, ♦), MOI 10 (gray, ×), and MOI 100 (yellow, ●). The bacterial culture not treated with bacteriophage (blue, ■) was used as a positive control. Optical density at 600 nm was measured hourly for 24 hours. Each data point represents the mean of 3 assays, each one with 3 replicas (n=9). Error bars represent standard errors based on the control series.

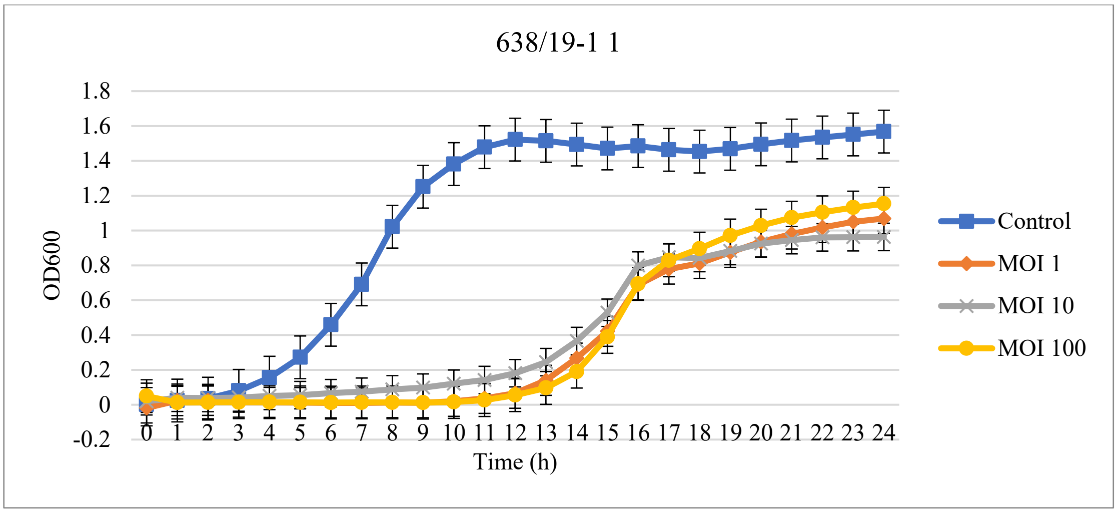

Figure S25- Time-kill assay of bacteriophage JG024 against *P. aeruginosa* 638/19-1 1 isolate. The bacterial culture was infected with phage JG024 at MOI 1 (orange, ♦), MOI 10 (gray, ×), and MOI 100 (yellow, ●). The bacterial culture not treated with bacteriophage (blue, ■) was used as a positive control. Optical density at 600 nm was measured hourly for 24 hours. Each data point represents the mean of 3 assays, each one with 3 replicas (n=9). Error bars represent standard errors based on the control series.

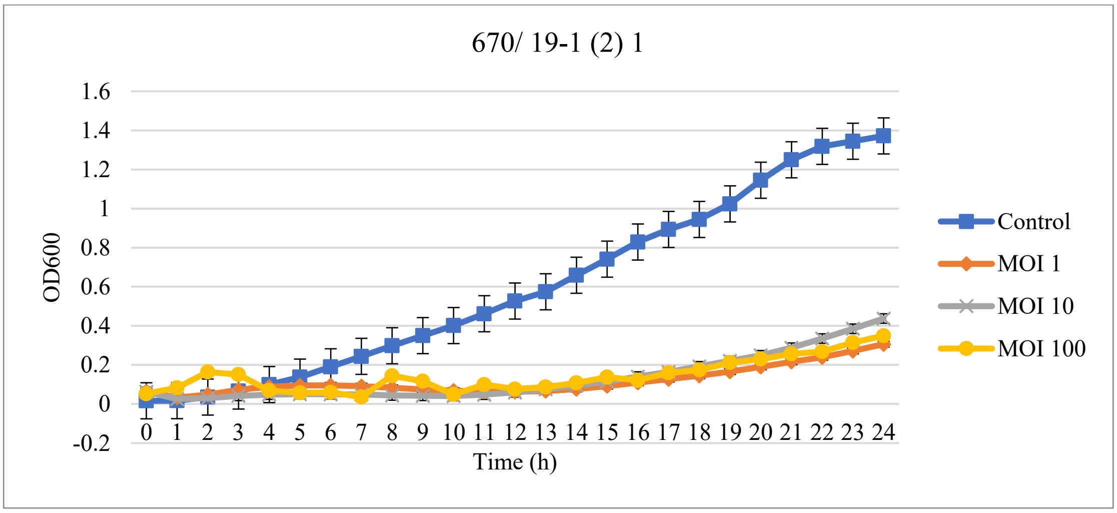

Figure S26- Time-kill assay of bacteriophage JG024 against *P. aeruginosa* 670/ 19-1 (2) 1 isolate. The bacterial culture was infected with phage JG024 at MOI 1 (orange, ♦), MOI 10 (gray, ×), and MOI 100 (yellow, ●). The bacterial culture not treated with bacteriophage (blue, ■) was used as a positive control. Optical density at 600 nm was measured hourly for 24 hours. Each data point represents the mean of 3 assays, each one with 3 replicas (n=9). Error bars represent standard errors based on the control series.

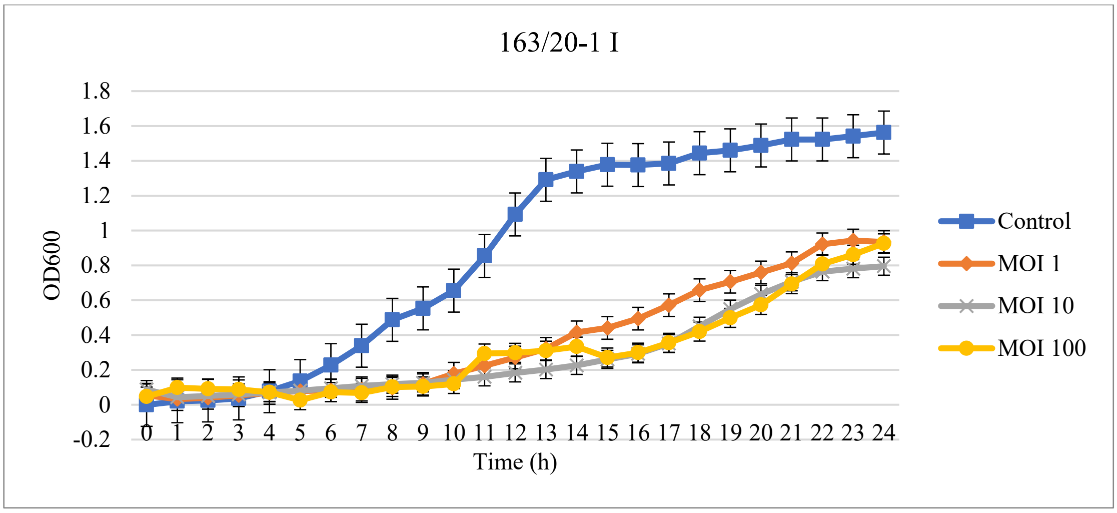

Figure S27- Time-kill assay of bacteriophage JG024 against *P. aeruginosa* 163/20-1 I isolate. The bacterial culture was infected with phage JG024 at MOI 1 (orange, ♦), MOI 10 (gray, ×), and MOI 100 (yellow, ●). The bacterial culture not treated with bacteriophage (blue, ■) was used as a positive control. Optical density at 600 nm was measured hourly for 24 hours. Each data point represents the mean of 3 assays, each one with 3 replicas (n=9). Error bars represent standard errors based on the control series.

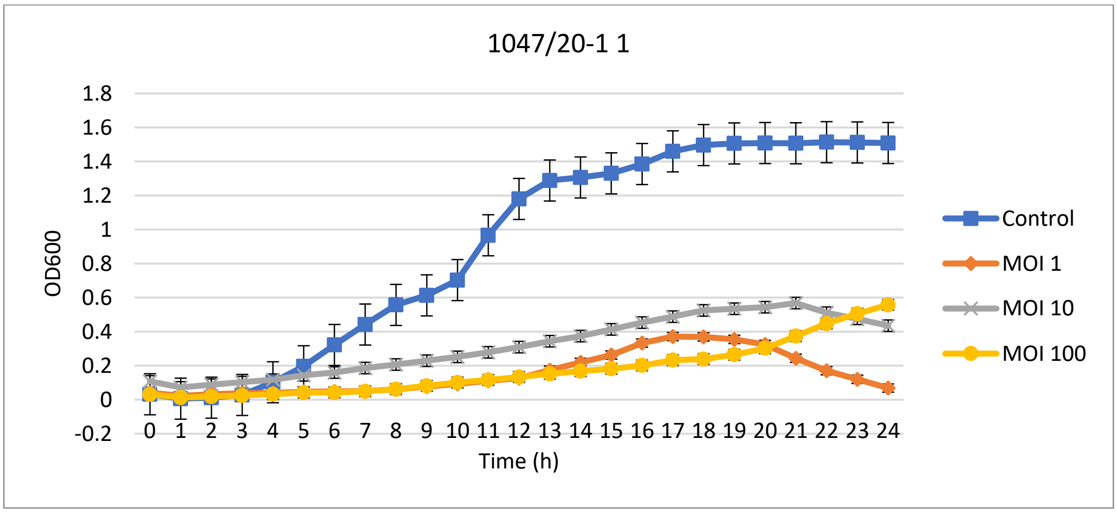

Figure S28- Time-kill assay of bacteriophage JG024 against *P. aeruginosa* 1047/20-1 1 isolate. The bacterial culture was infected with phage JG024 at MOI 1 (orange, ♦), MOI 10 (gray, ×), and MOI 100 (yellow, ●). The bacterial culture not treated with bacteriophage (blue, ■) was used as a positive control. Optical density at 600 nm was measured hourly for 24 hours. Each data point represents the mean of 3 assays, each one with 3 replicas (n=9). Error bars represent standard errors based on the control series.

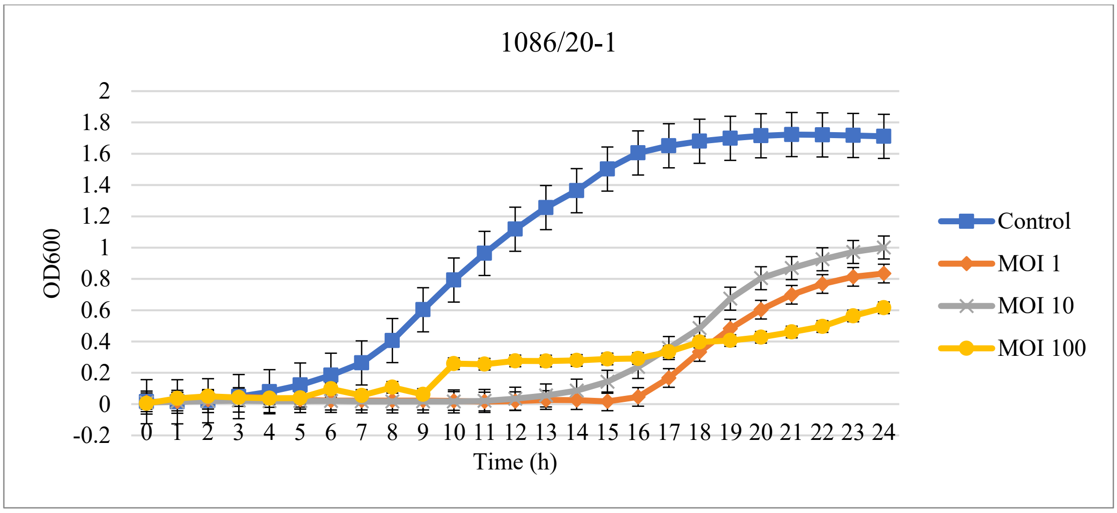

Figure S29- Time-kill assay of bacteriophage JG024 against *P. aeruginosa* 1086/20-1 isolate. The bacterial culture was infected with phage JG024 at MOI 1 (orange, ♦), MOI 10 (gray, ×), and MOI 100 (yellow, ●). The bacterial culture not treated with bacteriophage (blue, ■) was used as a positive control. Optical density at 600 nm was measured hourly for 24 hours. Each data point represents the mean of 3 assays, each one with 3 replicas (n=9). Error bars represent standard errors based on the control series.

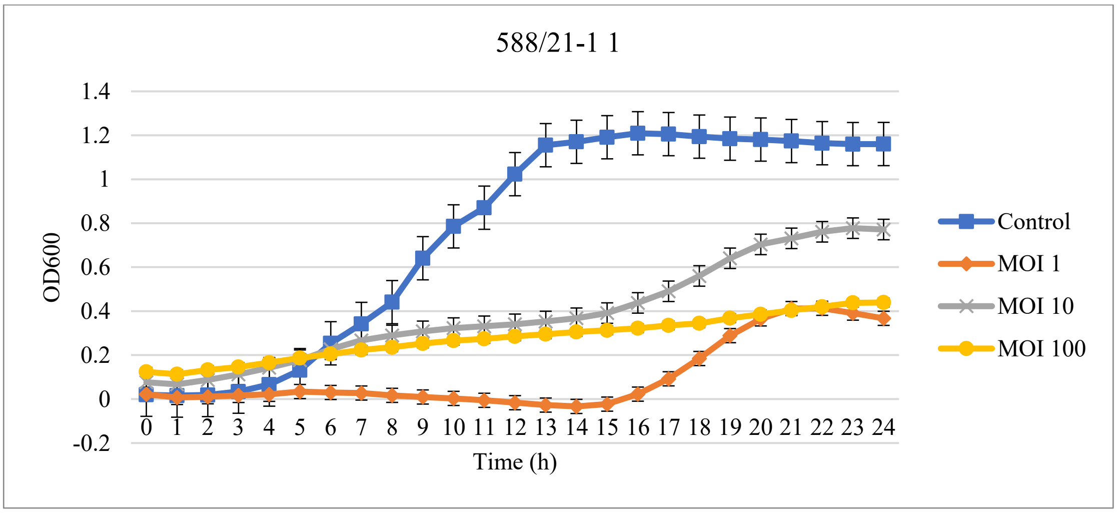

Figure S30- Time-kill assay of bacteriophage JG024 against *P. aeruginosa* 588/21-1 1 isolate. The bacterial culture was infected with phage JG024 at MOI 1 (orange, ♦), MOI 10 (gray, ×), and MOI 100 (yellow, ●). The bacterial culture not treated with bacteriophage (blue, ■) was used as a positive control. Optical density at 600 nm was measured hourly for 24 hours. Each data point represents the mean of 3 assays, each one with 3 replicas (n=9). Error bars represent standard errors based on the control series.

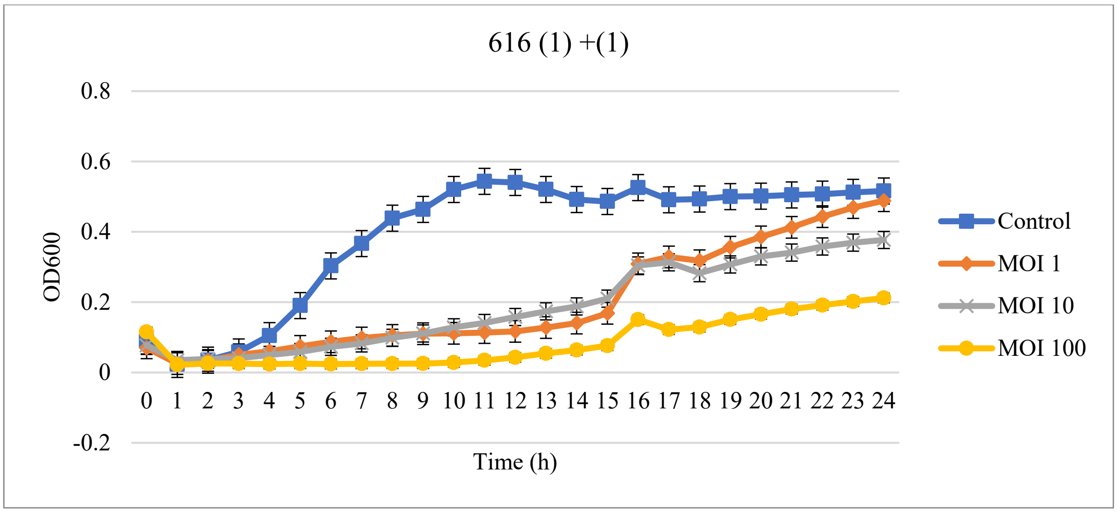

Figure S31- Time-kill assay of bacteriophage JG024 against *P. aeruginosa* 616 (1) +(1) isolate. The bacterial culture was infected with phage JG024 at MOI 1 (orange, ♦), MOI 10 (gray, ×), and MOI 100 (yellow, ●). The bacterial culture not treated with bacteriophage (blue, ■) was used as a positive control. Optical density at 600 nm was measured hourly for 24 hours. Each data point represents the mean of 3 assays, each one with 3 replicas (n=9). Error bars represent standard errors based on the control series.

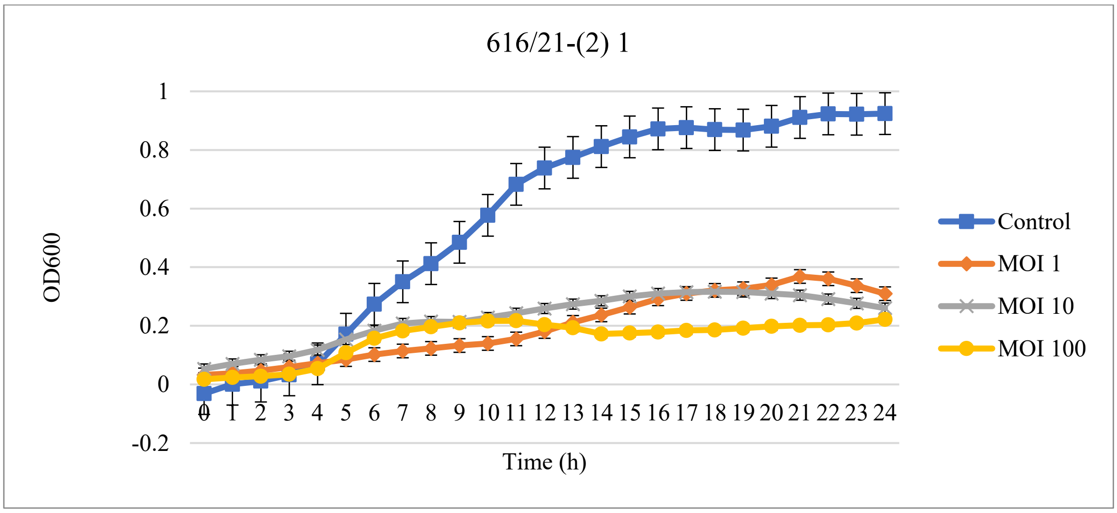

Figure S32- Time-kill assay of bacteriophage JG024 against *P. aeruginosa* 616/21-(2) 1 isolate. The bacterial culture was infected with phage JG024 at MOI 1 (orange, ♦), MOI 10 (gray, ×), and MOI 100 (yellow, ●). The bacterial culture not treated with bacteriophage (blue, ■) was used as a positive control. Optical density at 600 nm was measured hourly for 24 hours. Each data point represents the mean of 3 assays, each one with 3 replicas (n=9). Error bars represent standard errors based on the control series.

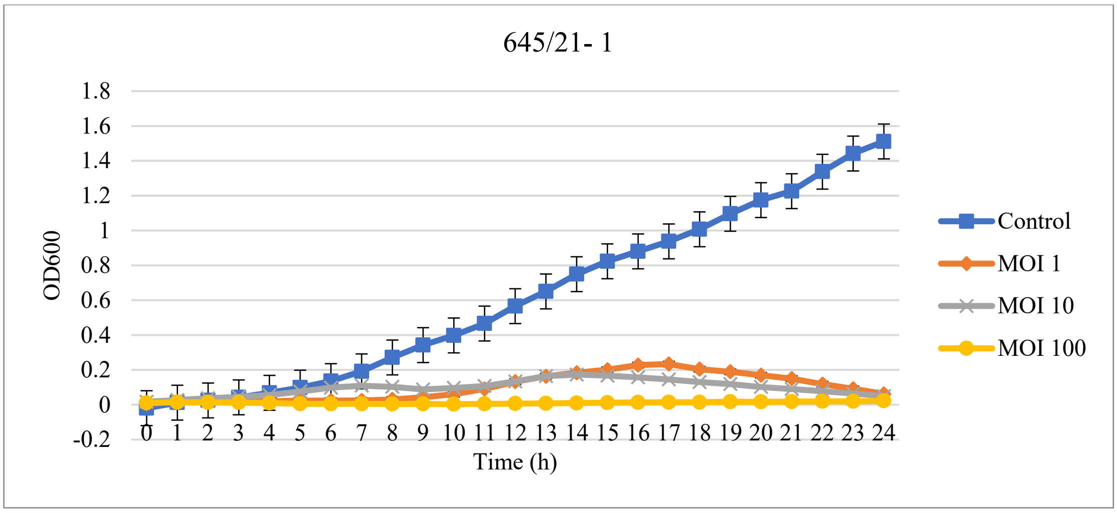

Figure S33- Time-kill assay of bacteriophage JG024 against *P. aeruginosa* 645/21- 1 isolate. The bacterial culture was infected with phage JG024 at MOI 1 (orange, ♦), MOI 10 (gray, ×), and MOI 100 (yellow, ●). The bacterial culture not treated with bacteriophage (blue, ■) was used as a positive control. Optical density at 600 nm was measured hourly for 24 hours. Each data point represents the mean of 3 assays, each one with 3 replicas (n=9). Error bars represent standard errors based on the control series.

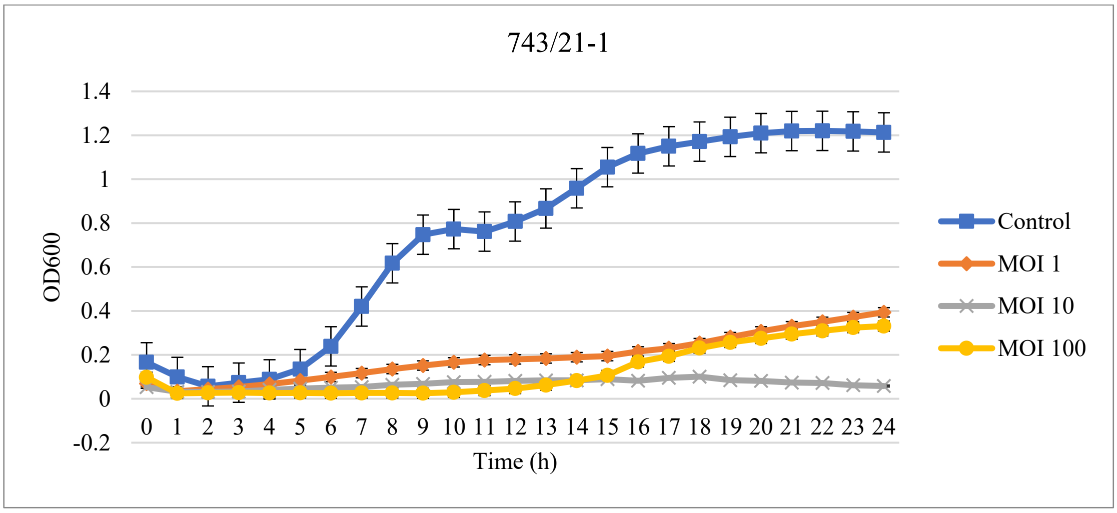

Figure S34- Time-kill assay of bacteriophage JG024 against *P. aeruginosa* 743/21-1 isolate. The bacterial culture was infected with phage JG024 at MOI 1 (orange, ♦), MOI 10 (gray, ×), and MOI 100 (yellow, ●). The bacterial culture not treated with bacteriophage (blue, ■) was used as a positive control. Optical density at 600 nm was measured hourly for 24 hours. Each data point represents the mean of 3 assays, each one with 3 replicas (n=9). Error bars represent standard errors based on the control series.

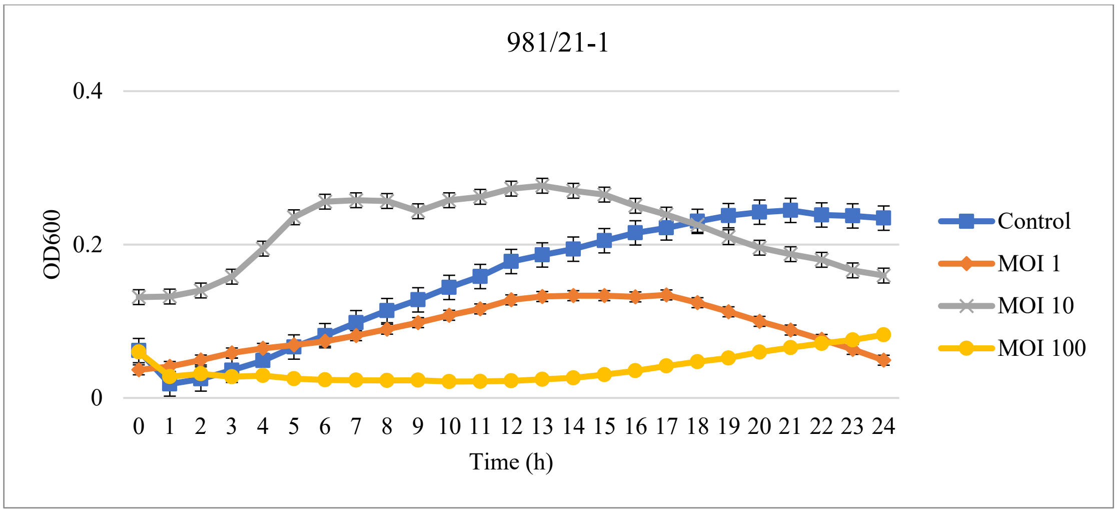

Figure S35- Time-kill assay of bacteriophage JG024 against *P. aeruginosa* 981/21-1 isolate. The bacterial culture was infected with phage JG024 at MOI 1 (orange, ♦), MOI 10 (gray, ×), and MOI 100 (yellow, ●). The bacterial culture not treated with bacteriophage (blue, ■) was used as a positive control. Optical density at 600 nm was measured hourly for 24 hours. Each data point represents the mean of 3 assays, each one with 3 replicas (n=9). Error bars represent standard errors based on the control series.

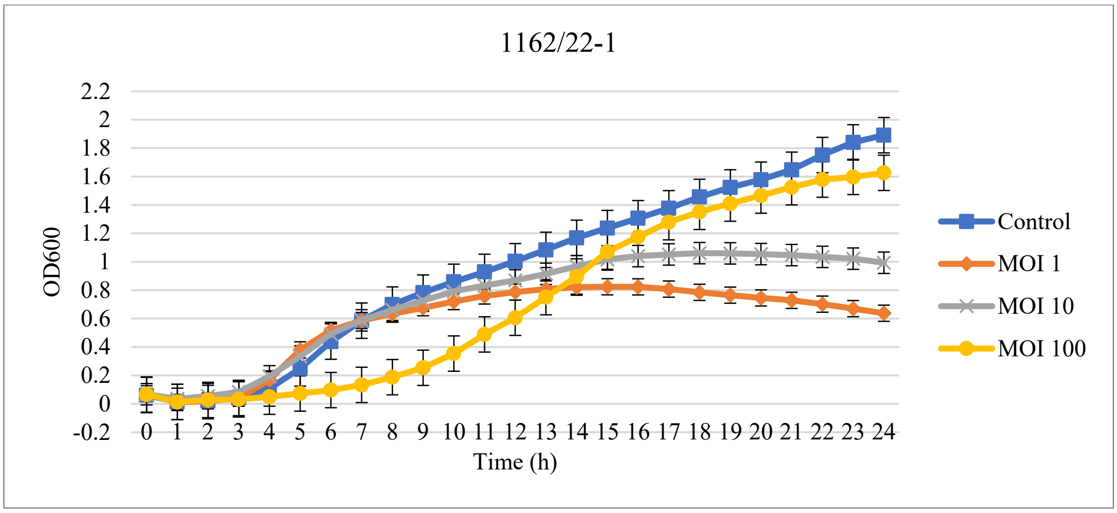

Figure S36- Time-kill assay of bacteriophage JG024 against *P. aeruginosa* 1162/22-1 isolate. The bacterial culture was infected with phage JG024 at MOI 1 (orange, ♦), MOI 10 (gray, ×), and MOI 100 (yellow, ●). The bacterial culture not treated with bacteriophage (blue, ■) was used as a positive control. Optical density at 600 nm was measured hourly for 24 hours. Each data point represents the mean of 3 assays, each one with 3 replicas (n=9). Error bars represent standard errors based on the control series.

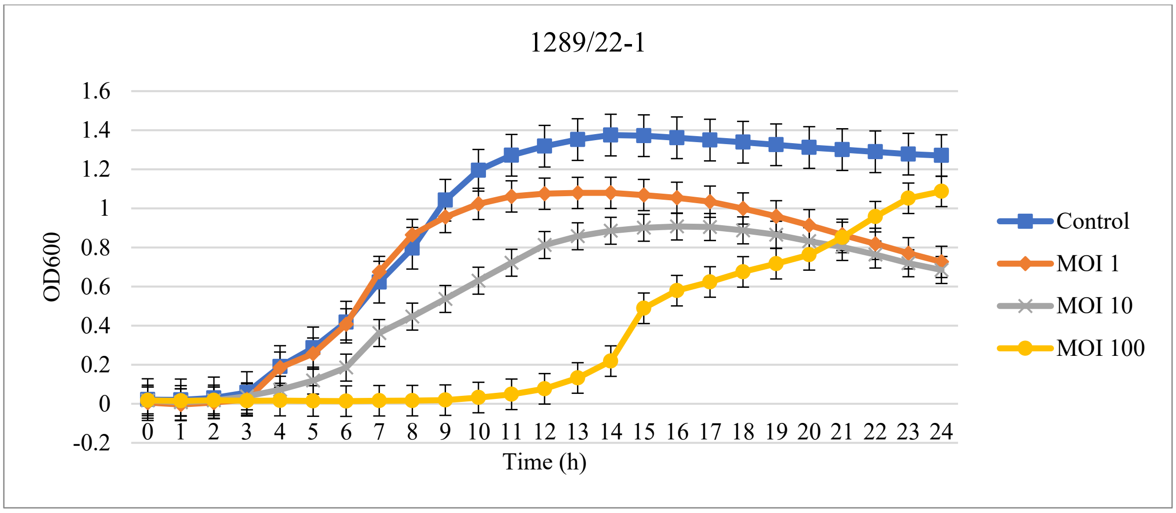

Figure S37- Time-kill assay of bacteriophage JG024 against *P. aeruginosa* 1289/22-1 isolate. The bacterial culture was infected with phage JG024 at MOI 1 (orange, ♦), MOI 10 (gray, ×), and MOI 100 (yellow, ●). The bacterial culture not treated with bacteriophage (blue, ■) was used as a positive control. Optical density at 600 nm was measured hourly for 24 hours. Each data point represents the mean of 3 assays, each one with 3 replicas (n=9). Error bars represent standard errors based on the control series.

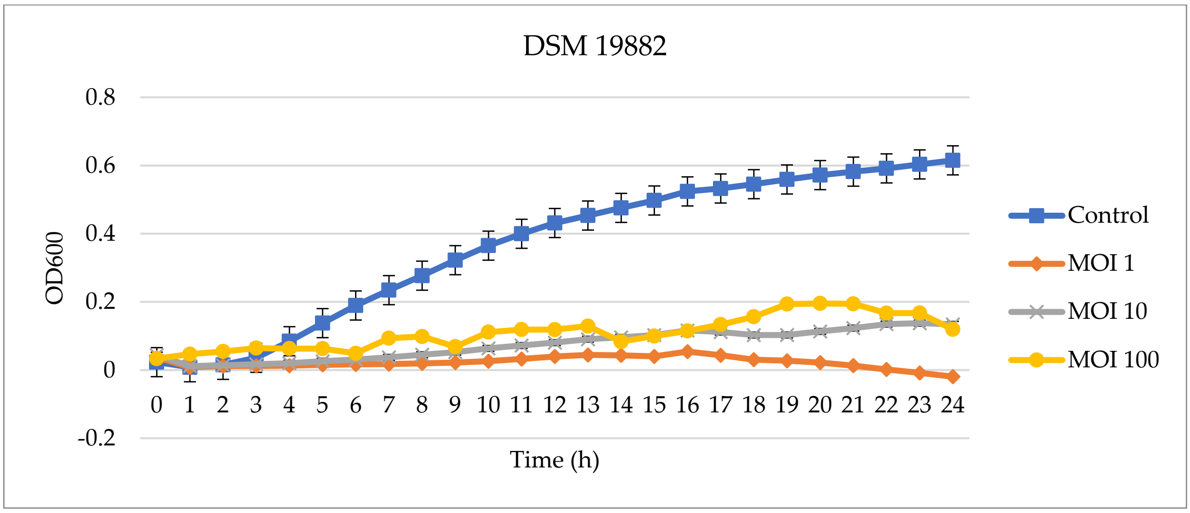

Figure S38- Time-kill assay of bacteriophage JG024 against *P. aeruginosa* DSM 19882. The bacterial culture was infected with phage JG024 at MOI 1 (orange, ♦), MOI 10 (gray, ×), and MOI 100 (yellow, ●). The bacterial culture not treated with bacteriophage (blue, ■) was used as a positive control. Optical density at 600 nm was measured hourly for 24 hours. Each data point represents the mean of 16 assays, each one with 3 replicas (n=48). Error bars represent standard errors based on the control series.
